# Documenting adaptations across the Accelerating Colorectal Cancer Screening and follow-up through Implementation Science research programs: methods and adaptation examples

**DOI:** 10.3389/frhs.2025.1613925

**Published:** 2025-09-26

**Authors:** Borsika A. Rabin, Erin S. Kenzie, Jill M. Oliveri, Aaron J. Kruse-Diehr, Sonja Hoover, Usha Menon, Mark P. Doescher, Prajakta Adsul, Shiraz I. Mishra, Kevin English, Jesse Nodora, Helen Lam, Karen Kim, Jennifer K. Coury, Melinda M. Davis, Teri Malo, Sarah Kobrin, Sujha Subramanian, Renée M. Ferrari

**Affiliations:** ^1^ Herbert Wertheim School of Public Health and Human Longevity Science, University of California San Diego, La Jolla, CA, United States; ^2^ UC San Diego Altman Clinical and Translational Research Institute Dissemination and Implementation Science Center, University of California San Diego, La Jolla, CA, United States; ^3^ Oregon Rural Practice-based Research Network, Oregon Health & Science University, Portland, OR, United States; ^4^ OHSU-PSU School of Public Health, Oregon Health & Science University, Portland, OR, United States; ^5^ Department of Population Sciences and Community Outreach, The Ohio State University Comprehensive Cancer Center, Columbus, OH, United States; ^6^ Department of Family and Community Medicine, University of Kentucky College of Medicine, Lexington, KY, United States; ^7^ Implenomics, Dover, DE, United States; ^8^ The University of South Florida and Tampa General Hospital Cancer Institute, Tampa, FL, United States; ^9^ Department of Family and Preventive Medicine and Stephenson Cancer Center, University of Oklahoma Health Sciences Center, Oklahoma City, OK, United States; ^10^ University of New Mexico Comprehensive Cancer Center, University of New Mexico Health Sciences Center, Albuquerque, NM, United States; ^11^ Department of Internal Medicine, University of New Mexico Health Sciences Center, Albuquerque, NM, United States; ^12^ University of New Mexico Comprehensive Cancer Center and Department of Pediatrics, University of New Mexico Health Sciences Center, Albuquerque, NM, United States; ^13^ Albuquerque Area Southwest Tribal Epidemiology Center, Albuquerque Area Indian Health Board, Inc., Albuquerque, NM, United States; ^14^ UC San Diego Health Moores Cancer Center, University of California San Diego, La Jolla, CA, United States; ^15^ Center for Asian Health Equity, University of Chicago, Chicago, IL, United States; ^16^ Department of Family Medicine, Oregon Health & Science University, Portland, OR, United States; ^17^ Lineberger Comprehensive Cancer Center, The University of North Carolina at Chapel Hill, Chapel Hill, NC, United States; ^18^ Healthcare Delivery Research, National Cancer Institute, Bethesda, MD, United States

**Keywords:** adaptation, implementation science, implementation strategies, mixed methods, colorectal cancer screening, data harmonization

## Abstract

**Introduction:**

Adaptations are common, expected, and often imperative for successful uptake and sustained implementation of clinical or public health programs in real-world practice settings. Understanding which adaptations have been made to evidence-based interventions and subsequent implementation strategies throughout the life cycle of a project can contextualize findings and support future scale-up of the program. Systematic documentation of adaptations is rarely conducted or reported, and little guidance exists on approaches to documenting adaptations.

**Methods:**

Accelerating Colorectal Cancer Screening and follow-up through Implementation Science (ACCSIS) is a National Cancer Institute-funded Beau Biden Cancer Moonshot^SM^ Initiative developed to improve colorectal cancer screening, follow-up, and referral for care among underserved groups, including diverse racial and ethnic populations and people living in rural areas. Using an iterative data gathering approach—a survey, data abstraction, and data validation—we compiled information about adaptation documentation and analytic methods and intervention and implementation strategy adaptations from the eight funded ACCSIS research programs. An analytic team representing multiple ACCSIS programs reviewed, coded, and summarized the data using a rapid qualitative analytic approach.

**Results:**

ACCSIS programs varied substantially in how they defined and documented adaptations. Nine approaches were used to document adaptations; the most common were periodic reflections and review of meeting minutes and agendas. Nine analytic methods were reported to guide adaptation analysis; the most frequently mentioned were rapid qualitative methods, descriptive statistics, and mixed-methods analysis. A total of 96 adaptations were reported by the eight research programs, most of which occurred during the pre-implementation stage (68%) or were made to the program format (71%). Only 36% of the adaptations were due to the COVID-19 pandemic.

**Conclusions:**

Our multi-method, systematic approach allowed us to explore how sites document and analyze adaptations across eight ACCSIS Moonshot programs. Using a systematic approach allowed for comparisons of intervention and strategy adaptations within and across research programs and can inform the science of adaptations, while building a knowledge base of why such adaptations are needed and how they can inform implementation efforts across time. Methods described herein provide a template for similar assessment activities in other large, multi-site research initiatives.

## 1 Introduction

Adaptations—defined as changes to an intervention or implementation strategy to increase fit to the context—are common, expected, and often necessary for the successful uptake and initial, ongoing, and sustained implementation of a program in a real-world setting (1). Understanding what adaptations have been made to both evidence-based interventions and implementation strategies throughout the life cycle of a study can help with the interpretation of findings, inform needed refinements, and support future scaling of the program in different settings (2–4). Systematic documentation of adaptations has not been conducted broadly and is a great need for the field of implementation science (5, 6). Furthermore, optimal approaches to documenting adaptations in complex studies and/or across multi-site initiatives and how to assess the impact of these adaptations on key implementation and effectiveness outcomes are still not well understood (7, 8).

Although comprehensive frameworks exist to guide the process of adaptation in the pre-implementation phase (9, 10) and to provide a nomenclature of the type of adaptations to interventions and implementation strategies (11, 12), guidance is lacking on how to collect and analyze data about adaptations in the context of research studies. There is a growing consensus that more than one method should be used to document adaptations; however, it is unclear what combination of methods for documenting adaptations yields the most meaningful information (13). There is even less guidance on how to assess the impact of adaptations on diverse implementation outcomes.

Finally, using systematic approaches to document adaptations across multiple research programs (RPs) longitudinally would allow for comparison of intervention and strategy adaptations both within each RP and across RPs over time. This approach can also provide a template for adaptation documentation and assessment activities in other large research initiatives.

To address these needs, we aimed to describe (1) the process of developing a cross-RP documentation system for a large consortium, (2) the methods used for tracking intervention and strategy adaptations across RPs, (3) barriers and facilitators to implementing those methods, and (4) potential value to advance implementation science. We review and summarize adaptations made in pre-, early-, and mid-implementation phases and discuss next steps to refine the tracking system and process moving forward.

## 2 Methods

### 2.1 The ACCSIS consortium

This study was conducted as part of the National Cancer Institute-funded Beau Biden Cancer Moonshot^SM^ Initiative (14). The overall aim (15) of ACCSIS is to conduct multisite, coordinated, transdisciplinary research to evaluate and improve colorectal cancer (CRC) screening processes using implementation science.

NCI began funding ACCSIS RP in 2018 to improve CRC screening, follow-up, and referral for care among populations that have low CRC screening rates (16), with a focus on underserved groups including diverse racial and ethnic populations and people living in rural areas. The initiative comprises a multisite consortium that includes a Coordinating Center (Research Triangle Institute—RTI) and eight RPs located across the United States and working with diverse populations including American Indian, other racial and ethnic minorities, rural populations, and people who are uninsured. Additional File S1 provides an overview of the key objectives, geographical locations, and populations served for the eight ACCSIS RPs.

### 2.2 Origins of adaptation documentation in ACCSIS

The objectives and methods for the work presented in this manuscript emerged from a series of Intervention, Strategies, and Adaptations Workgroup meetings conducted between summer 2020 and fall 2021 involving representatives from all eight ACCSIS RPs. Within the larger ACCSIS initiative, the workgroup's purpose is to contribute to the development of effective and sustainable multilevel interventions and strategies across all RPs. The workgroup identified documentation of adaptations as a shared interest and engaged in a series of consensus discussions, reviewed the literature, and invited experts in the field to present about their experience in documenting adaptations. Although there was initial variation in the level of interest and expertise across the RPs regarding documenting and analyzing adaptations, all RPs ultimately agreed to document adaptations. Data collection about adaptations and documentation methodology was conducted cross-sectionally starting in fall 2021.

### 2.3 Data collection and analysis for methods to document and analyze adaptations

To understand how individual RPs defined adaptations, collected information about adaptations, and intended to analyze the adaptations, we developed an instrument based on prior publications by some of the authors of this manuscript (BAR, ESK, JKC, MMD) along with input from all members of a smaller writing group representing four of the RPs (California, Kentucky, North Carolina, Oregon), NCI, and RTI. The final version of the data collection instrument is available as Additional File S2 and includes 13 questions about how adaptations were defined; who decided about what changes to the intervention, implementation strategies, and RP were considered adaptations; theoretical frameworks guiding adaptation documentation and analysis; processes and methods used to document adaptations (i.e., how, who, from whom, using what type of data, how frequently, what type of information); and methods planned for analysis of adaptations. Data from the surveys were summarized by RP and across programs using descriptive statistics (frequencies and averages).

### 2.4 Documenting and reviewing adaptations

To operationalize our adaptation documentation approach, we asked each RP to share a list of adaptations made to their intervention or implementation strategies, from program inception until a cut-off date selected (i.e., all studies were in the pre- to mid-implementation phase at the time of the request) by the working group for the purpose of bounding data collection. The intention was not to collect a complete list of adaptations but instead to assess the feasibility of collating adaptations from the eight RPs. Throughout the manuscript, we refer to these sample adaptations simply as “adaptations” for clarity and ease of reporting. Adaptations were classified as occurring during the pre-implementation, early-implementation, or mid-implementation phases by each program. Pre-implementation indicated an adaptation that happened prior to the delivery of the intervention (e.g., prior to patient list pulled by care coordinator). Early implementation was classified as during early implementation activities (e.g., post identification of eligible patient list but prior to mailing the test kits), while mid-implementation was defined as during the more advanced implementation of the program (i.e., post mailing the test kits).

Adaptation data were collected from RPs and then collated and organized in an Excel database. Each RP sent the following information about their adaptations: (1) what the adaptations were, (2) when they were made, (3) the reasons for the adaptations, (4) what component(s) of the program were adapted, and 5) whether the adaptations were related to the COVID-19 pandemic. The collated adaptations from all RPs were then reviewed for consistency, and additional coding was undertaken by a small analytic team (BAR, ESK, JMO, AK-D, RMF) to identify sub-categories for adaptation description and why the adaptation was made. The workgroup coded the adaptation data using FRAME: an expanded framework for reporting adaptations and modifications to evidence-based interventions (11). We decided to use a subset of the FRAME constructs instead of the full set of constructs for our combined database to make the documentation process more pragmatic and accessible to all participating RPs. We used a team-based approach to coding where at least two coders reviewed each adaptation entry and reached consensus using a shared codebook regarding the appropriate category. Instead of calculating inter-coder agreement, we used a consensus approach to resolve disagreement in coding. The coded adaptations were then returned to each RP for member-checking (17, 18), and refinements and modifications were incorporated based on feedback from the RPs. Characteristics of adaptations were then summarized using descriptive statistics.

## 3 Results

Findings are summarized in two sections. First, we summarize information about methods used across the eight RPs to document and analyze adaptations (Tables 1 through 3B). Next, we describe findings from the compilation and coding of the adaptations shared with the workgroup by all RPs (Table 4 and Additional File S3).

**Table 1:** Adaptation definitions and personnel defining adaptations by ACCSIS research.

ACCSIS research project	Adaptation definition	Who decides about what is an adaptation?^a^	Theoretical framework guiding adaptation documentation and analysis
Appalachia	Anything that is different from either (1) the original evidence-based intervention; or (2) what was planned by the site champion and the implementation team.	Researcher(s)/Research Team Implementation partners	None
Arizona	Community-engaged adaptation for cultural sensitivity; response to circumstances that were not anticipated in the pre-implementation planning phase.	Researcher(s)/Research Team Implementation partners	7-step framework (19)
Chicago	Adaptation is a thoughtful process of identifying discordance between the intervention and the targeted context or population and a deliberative alteration to an intervention to improve its effectiveness.	Researcher(s)/Research Team Implementation partners	DSF (3)^z^
New Mexico	Changes to intervention strategies based on unique population context and settings, as guided by multisector action team which guides the implementation process at each healthcare facility; includes both COVID and non- COVID changes.	Researcher(s)/Research Team	None
North Carolina	Any change made to the planned intervention or planned implementation strategies during the pilot or trial period.	Researcher(s)/Research Team	FRAME (11)^x^ FRAME-IS (21)^y^
Oklahoma	Any change made to the original interventions and implementation strategies. Also, unexpected changes in staffing (e.g., navigator turnover due to COVID), which is not a change to the original protocol but has created challenges to the ongoing implementation of interventions.	Researcher(s)/Research Team Implementation partners	None
Oregon	Any change to interventions and implementation strategies (not including context or disruptions or research methods changes).	Researcher(s)/Research Team	FRAME (11)^x^ iPARIHS (20)
San Diego	Any change to the program planned or unplanned compared to what was initially planned, implementation strategies, context, or research methodology (e.g., design, measures, recruitment, inclusion criteria, etc).	Researcher(s)/Research Team Research and implementation facilitation organization	RE-AIM expanded FRAME (13)^v^

^a^ Answer options included: Researcher(s)/Research Team; Implementation partners (clinic administrators, program managers, clinicians, healthcare staff, etc.); Community members/Patients; Other (specify).

DSF, dynamic sustainability framework; FRAME, framework for reporting adaptations and modifications to evidence-based interventions; FRAME-IS, framework for reporting adaptations and modifications to evidence-based implementation strategies; iPARIHS, integrated promoting action on research implementation in health services, RE-AIM: reach, effectiveness, adoption, implementation, maintenance framework.

### 3.1 Approaches to defining and capturing adaptations

ACCSIS RPs varied substantially in how they approached capturing adaptations to their intervention and implementation strategies. Some programs used a comprehensive approach to collect a variety of data, using multiple methods guided by a framework, while others collected less comprehensive adaptation information.

Table 1 provides an overview of each RP's definitions of adaptations, the teammates who decided what constituted an adaptation to their program, and what (if any) frameworks were used to guide adaptation documentation and analysis. Adaptation definitions included broad, inclusive definitions such as “Response to circumstances that were not anticipated in pre-implementation planning phase” (ACCSIS Arizona), as well as more specific definitions: “Changes to intervention strategies based on unique population context and settings, as guided by multisector action team which guides the implementation process at each health care facility; includes both COVID and non-COVID changes” (ACCSIS New Mexico). Most definitions included the concept of improving fit to context (setting, population), and multiple definitions included reference to both intervention and implementation strategy. The role of the COVID-19 pandemic was explicitly mentioned in two definitions.

Five of the eight RPs relied on expertise from the research team and either implementation partners or research and implementation partners in deciding what counts as an adaptation; three RPs primarily relied on the research team for these decisions. Over half of the RPs reported the use of one or more frameworks to guide the documentation and analysis of adaptations. Frameworks that were identified included 7-step adaptation framework (19), Framework for Reporting Adaptations and Modifications to Evidence-based Interventions (FRAME) (*n* = 2) (11), Framework for Reporting Adaptations and Modifications to Evidence-based Implementation Strategies (FRAME-IS) (20), RE-AIM (Reach, Effectiveness, Adoption, Implementation, and Maintenance)-expanded FRAME (13), Integrated Promoting Action on Research Implementation in Health Services (iPARIHS) (21), and the Dynamic Sustainability Framework (DSF) (3).

### 3.2 Methods for documenting and analyzing adaptations

Table 2 displays the great diversity in the number and type of methods that RPs used for the documentation, analysis, and operationalization of adaptations, stratified by RP. Tables 3A, 3B provide a summary of the Table 2 data, organized by adaptation characteristic (e.g., method, analysis plan, data collection frequency). Specifically, Table 3A summarizes the methods used and planned analyses, and Table 3B summarizes how the RPs operationalized their methods.

**Table 2:** Key characteristics of adaptation documentation by ACCSIS research program.

ACCSIS research project	Methods to document adaptation data	Operationalization of method	Who collects the data?	From whom data is being collected	Type of data collected	Frequency for data collection	Adaptation information collected/coded**^a^**	Methods for analyzing adaptation data
	What methods are used to collect the adaptation data? (Select all that apply)	How do you operationalize this method	Who collects the data		What types of data are collected about the adaptation? (Select all that apply)	How frequently are adaptation data collected?	What information is collected about the adaptations? (Select all that apply)	How will you analyze/are you analyzing the adaptation information? Please consider your analytic approaches across all adaptation documentation methods. (Select all that apply)
Appalachia	Adaptations tracking at clinical site	Clinic champions describe any planned or unplanned adaptations during monthly and quarterly check-in calls.	Researcher(s)/Research Team: Project manager, research team	Implementation partners: Clinic champions and implementation team members	QUAL QUANT	Monthly Quarterly Variable (as needed/*ad hoc*)	ALL	Rapid qualitative analysis Descriptive statistics
	Review of meeting minutes and agendas	Project team members review minutes from monthly and quarterly clinic check-in calls to review any updates, including adaptations.	Researcher(s)/Research Team: Project manager, research team	Implementation partners: Clinic champions and implementation team members	QUAL QUANT	Monthly Quarterly	ALL	
	Informal check-in with clinical or community partners	Clinic champions describe any planned or unplanned adaptations during progress check-ins with project manager.	Researcher(s)/Research Team: *Project manager*	Implementation partners: *Clinic champions*	QUAL QUANT	Variable (as needed, *ad hoc*)	ALL	
Arizona	Periodic reflections for research team and/or implementation partners	Combination of weekly and monthly in person and virtual meetings with navigators at clinical facilities, research team, and key personnel at all sites	Researcher(s)/Research Team: Data analyst and administrator with supervision from research PIs	Researcher(s)/Research Team: *All members* Implementation partners: Medical providers, clinic administrators, navigators	QUAL QUANT	Real time	ADAPT TYPE DESC SOURCE ELEMENTS WHEN WHO WHY* IMPACT	Standard/traditional (in-depth) qualitative analysis TBD
Chicago	Adaptations tracking at clinical site	Track the intervention adaptation at each partner health system based on five domains: Service setting Target audience Mode of delivery Cultural Core component	Researcher(s)/Research Team: *Project manager*	Implementation partners	QUAL QUANT	Variable (as needed, *ad hoc*)	ADAPT TYPE PLAN DESC SOURCE ELEMENTS CHANGE TYPE WHEN WHY IMPACT	Analyses to examine impact of adaptations on various outcomes
	Review clinic contact logs and field notes	Feedback from implementer at partner site	Researcher(s)/Research Team: *Project manager*	Implementation partners: Program manager, navigator, QI manager	QUAL	Variable (as needed, *ad hoc*)	ADAPT TYPE ELEMENTS CHANGE TYPE WHY	
New Mexico	Periodic reflections for research team and/or implementation partners	Research team reviewed research project and identified adaptations.	Researcher(s)/Research Team: Research team members including facilitators	Researcher(s)/Research Team: Research team members including facilitators	QUAL	Variable (as needed, *ad hoc*)	ADAPT TYPE DESC WHY BASIS	Mixed methods analysis
	Review of meeting minutes and agendas	Detailed notes for the meetings with the implementing partners (i.e., multisector action team) at the participating healthcare facilities are captured. All meeting notes are reviewed for strategies chosen and adaptation made.	Researcher(s)/Research Team: Research team members including facilitators	Researcher(s)/Research Team: Research team members including facilitators Implementation partners: Multisector action team at each participating health facility	QUAL	Monthly	ADAPT TYPE DESC ELEMENTS CHANGE TYPE WHEN WHO WHY IMPACT BASIS	
North Carolina	Interviews with implementers	Interviews with implementers are intended to gather interviewee perspectives on implementation and adaptations. The ACCSIS-NC (SCORE) Capturing Adaptations (CA) team identifies key clinic partners and research staff who are directly involved with the intervention. Two CA team members facilitate the interview using a semi-structured interview guide based on the FRAME. Interviews are audio-recorded and transcribed.	Researcher(s)/Research Team: Co-investigators, project managers, research assistants, patient navigator	Researcher(s)/Research Team: *Full research team* Implementation partners: Partner clinic practice managers, navigators, IT/data/clinical application specialists, quality improvement staff, population health director and managers, lab manager	QUAL	Variable (as feasible, *ad hoc*)	ADAPT TYPE PLAN SOURCE ELEMENTS CHANGE TYPE WHEN WHY IMPACT	Rapid qualitative analysis TBD
	Periodic reflections w/ clinical site implementers during standing meetings	Periodic reflections are intended to provide an opportunity for reflecting on implementation and adaptations on a regular basis. At the beginning of standing monthly (previously bimonthly) meetings with partner clinic staff, the CA teams asks about 3–5 semi-structured reflection questions developed by the team. Two SCORE members then take notes on the clinic's response and, for the rest of the meeting, listen for language about adaptions and note down any topics appropriately. These notes are abstracted into a spreadsheet organized by reflection question and a subset of FRAME domains.	Researcher(s)/Research Team: Co-investigators, project managers, research assistants, patient navigators	Implementation partners: Clinic administrators, chief medical officers, practice managers, quality improvement staff, navigators, IT/data/clinical application specialists, lab manager, support staff	QUAL	Bi-weekly Monthly	ADAPT TYPE PLAN* DESC SOURCE ELEMENTS CHANGE TYPE* WHEN WHO WHY IMPACT* BASIS*	
	Periodic reflections with research team	Because our project is centralized, research team members are also implementers. Reflections with the research team focus on each intervention component (Registry, Mailed FIT, FIT+ to Colonoscopy) and allow the team to collectively reflect on and develop shared understanding of implementation activities and adaptations.	Researcher(s)/Research Team: Co-investigators, project managers, research assistants, patient navigators	Researcher(s)/Research Team: *Full research team*	QUAL	Variable (as feasible, *ad hoc*)	ADAPT TYPE PLAN* DESC SOURCE ELEMENTS CHANGE TYPE* WHEN WHO WHY IMPACT* BASIS*	
	Review of meeting minutes and agendas	Using meeting notes from all project meetings, team will retrospectively code and analyze meeting notes, with specific focus on FRAME topics.	Researcher(s)/Research Team: Co-investigators, project managers, patient navigators research assistants	Researcher(s)/Research Team: Principal investigator, co-investigators, project managers, patient navigators research assistants Implementation partners: Clinic administrators, chief medical officers, practice managers, quality improvement staff, navigators, IT/data/clinical application specialists, lab manager, support staff; American Cancer Society (ACS) liaisons	QUAL	Weekly Bi-weekly Monthly Quarterly	ALL	
Oklahoma	Adaptations tracking at clinical site	Navigator tracking log is used to record number of FIT kits mailed/returned at each clinic per month, how the FIT kits were distributed and returned (e.g., by mail, at the clinic, etc). This allows us to determine how these numbers vary when adaptations are made, including standing orders for FIT tests, distribution of FIT tests at COVID-19 vaccine and influenza vaccine events, mailed FIT kits with return envelopes, and a monthly lottery for a $20 gift card for returning FIT kits at one site.	Researcher(s)/Research Team Implementation partners: *Navigators*	Implementation partners: *Navigators*	QUANT	Monthly	ADAPT TYPE DESCR SOURCE ELEMENTS CHANGE TYPE WHEN WHO WHY IMPACT BASIS	Descriptive statistics Content analysis
Oregon	Interviews with implementers	Semi-structured qualitative interviews with health plan and clinic partners. Research team prepares an interview guide and conducts the interviews in person or via zoom. Sessions are recorded and transcribed.	Researcher(s)/Research Team: Qualitative analysts, practice facilitators, project manager	Implementation partners: Primary care clinic staff and providers, health plan staff	QUAL	Other: At baseline, following Year 1 of implementation, post-implementation	ALL	Standard/traditional (in-depth) qualitative analysis Mixed methods analysis Analyses to examine impact of adaptations on various outcomes Causal loop modeling Member checking with local advisory board Configurational Comparative Methods (CCM) and Qualitative Comparative Analysis (QCA)
	Periodic reflections for research team and/or implementation partners	Qualitative analyst conducts monthly periodic reflection with practice facilitators and several supplemental periodic reflections with the whole research team at key points in the study. Qualitative analyst solicits topics from research team prior to session. Sessions are conducted via zoom, recorded, and transcribed.	Researcher(s)/Research Team: *Qualitative analyst*	Researcher(s)/Research Team: Practice facilitators (monthly), broader reflections include project manager, principal investigator, analysts, facilitators	QUAL	Monthly Other: Key points in study (following recruitment, after Year 1 implementation, post-implementation)	ALL	
	Review clinic contact logs and field notes	Facilitators complete field note forms in REDCap following clinic encounters. Forms include open and closed ended questions.	Researcher(s)/Research Team: *Practice facilitators*	Researcher(s)/Research Team: *Practice facilitators* Implementation partners: Clinic staff and providers	QUAL QUANT	Real time	ADAPT TYPE PLAN DESC SOURCE WHEN WHO WHY BASIS	
	Review of meeting minutes and agendas	Project manager and practice facilitators take notes to summarize meetings with health plan partners and clinic staff.	Researcher(s)/Research Team: Project manager, practice facilitators	Implementation partners: Clinic staff and providers, health plan staff	QUAL	Monthly Variable (as needed, *ad hoc*)	DESC SOURCE WHY	
	Member-checking of data for missing adaptations and accuracy of data	Adaptations are logged into a tracking spreadsheet and reviewed for accuracy with project leadership team (project manager, PIs, lead analysts).	Researcher(s)/Research Team: *Qualitative analyst*	Researcher(s)/Research Team: Project manager, practice facilitators, principal investigators, analysts Implementation partners: Clinic staff and providers, health plan staff	QUAL QUANT	Variable (as needed, *ad hoc*) Other: Data is compiled following qualitative analysis at key points in the study (baseline, following Year 1, post implementation)	ALL	
San Diego	Interviews with implementers	A series of semi-structured interviews will be conducted mid-implementation and at the end of implementation with CHC stakeholders (e.g., Care Coordinators, CHC leaders, and clinicians). The interviews will include adaptation related questions.	Researcher(s)/Research Team: Investigators and implementation science specialist	Implementation partners: Care coordinators and other key CHC staff	QUAL	Other: mid-implementation and end of implementation	DESC SOURCE WHEN WHO WHY IMPACT BASIS	Rapid qualitative analysis Descriptive statistics Content analysis Mixed methods analysis
	Periodic reflections for research team and/or implementation partners	During regular weekly team meetings, including the research team and research and implementation facilitation organization, discussions about challenges and potential solutions and related adaptations are conducted. Discussion is led by the implementation science specialist.	Researcher(s)/Research Team: Implementation science specialist	Researcher(s)/Research Team: *Entire team* Other: Research and implementation facilitation partner	QUAL	Variable (as needed, *ad hoc*)	DESC SOURCE ELEMENTS CHANGE TYPE WHEN WHO WHY IMPACT BASIS OTHER^b^	
	Real-time adaptation tracking database	Adaptation data is submitted by the team and manually recorded on a REDCap survey to enter into the adaptation-tracking database.	Researcher(s)/Research Team: Implementation science specialist (primarily), entire team Other: Research and implementation facilitation partner	Researcher(s)/Research Team: *Entire team* Implementation partners: CHC champions (e.g., care coordinators, lab supervisors) Other: Research and implementation facilitation partner	QUAL QUANT	Real time	DESC SOURCE ELEMENTS CHANGE TYPE WHEN WHO WHY IMPACT BASIS OTHER^b^	
	Reminder email about adaptations to research team and partners	The Project Manager disseminates biweekly reminder email sent to the research team and the research and implementation facilitation organization.	Researcher(s)/Research Team: Implementation science specialist	Researcher(s)/Research Team: *Entire team* Other: Research and implementation facilitation partner	QUAL	Bi-weekly	DESC OTHER^b^	
	Review of meeting minutes and agendas	Review of weekly meeting minutes, attended by the research team and the research and implementation facilitation organization, and meeting agendas to identify and capture adaptations.	Researcher(s)/Research Team: Implementation science specialist	Researcher(s)/Research Team: *Entire team* Other: Research and implementation facilitation partner	QUAL QUANT	Variable (as needed, *ad hoc*)	DESC SOURCE ELEMENTS CHANGE TYPE WHEN WHO WHY IMPACT BASIS OTHER^b^	
	Informal check-in with clinical or community partners	The research and implementation facilitation organization meets biweekly with each participating health center to discuss implementation and related challenges.	Other: Research and implementation facilitation partner	Implementation partners: Care coordinators and other key CHC staff	QUAL	Bi-weekly	DESC SOURCE ELEMENTS CHANGE TYPE WHEN WHO WHY IMPACT BASIS OTHER^b^	
	Member-checking of data for missing adaptations and accuracy of data	All adaptations document in the real-time adaptation database are presented to the research team and research and implementation facilitation organization to review and confirm details of adaptation and identify missing adaptations.	Researcher(s)/Research Team: Implementation science specialist	Researcher(s)/Research Team: *Entire team* Other: Research and implementation facilitation partner	QUAL QUANT	Semi-annually	DESC SOURCE ELEMENTS CHANGE TYPE WHEN WHO WHY IMPACT BASIS OTHER^b^	

^a^ Adaptation information could be either systematically collected or coded based on data available.

^b^ Other data about adaptations collected by San Diego included: Person entering adaptation into database, Site(s) where adaptations are relevant, Date adaptations entered into database, Name of adaptation, How was information on the adaptation documented, Position of the person providing information on adaptation, COVID-19 Impact, Study core components impacted, Who was primarily responsible for initiating modification, At which point in the project was this change FIRST made, Supporting documentation, Additional comments, Team member validation and date.

QUAL, qualitative data; QUANT, quantitative data.

ADAPT TYPE—Type of adaptation (e.g., change in process, strategy, intervention, etc.).

PLAN—Adaptation was planned or unplanned.

DESC—Description of adaptation.

ELEMENT—Elements of the program were changed (e.g., setting, format, personnel, etc.).

SOURCE—Who provided information about the adaptation.

CHANGE TYPE—Type of change was made (e.g., tailoring, adding component, etc.)?.

WHEN—When adaptation was made.

WHO—Who initiated the adaptation.

WHY—Why the adaptation was made.

IMPACT—What the impact of the adaptation was.

BASIS—What the basis for the change (e.g., vision or values, framework or theory, staff or patient knowledge, etc.) was.

Interviews: Purposeful, structured conversations using specific adaptation-related questions set up formally, usually recorded and transcribed, with those involved in the intervention and/or its implementation (including research team and/or formal partners). Interviews are unidirectional, with the interviewer asking questions/follow-up questions and the participant responding. The purpose is to understand participant thoughts and perspectives.

Periodic reflections: Periodic, guided discussions, with specific adaptations-related questions, may or may not be recorded and transcribed, with those involved in the intervention and/or its implementation (including research team and/or formal partners). The discussions are bi-directional, with shared learnings and understandings created through the conversation. The purpose is to reflect on the intervention, its implementation, and adaptations to develop shared understanding and learnings. [adapted from Finley et al. (22), Periodic reflections].

**Table 3A:** Summary characteristics of method used to document and analyze adaptations in the 8 ACCSIS research programs.

Adaptation characteristic	*n*/%
Methods to document adaptation data Total: 25 documentation method use/9 unique documentation methods reported Average number of methods per Research Program: 3.1 Range of number of methods: 1 and 7	• Periodic reflections for research team and/or implementation partners: 5 (6 with NC two types) • Review of meeting minutes and agendas: 5 • Interviews with implementers: 3 • Adaptations tracking at clinical site: 3 • Review clinic contact logs and field notes: 2 • Informal check-in with clinical or community partners: 2 • Member-checking of data for missing adaptations and accuracy of data: 2 • Real-time adaptation tracking database: 1 • Reminder email about adaptations to research team and partners: 1
Methods for analyzing adaptation data Total: 18 analysis method use/9 unique analysis methods reported Average per Research Program: 2.25 Range: 1 and 6	• Rapid qualitative analysis: 3 • Descriptive statistics (e.g., mean, median, mode, frequency, range): 3 • Mixed methods analysis: 3 • Standard/traditional (in-depth) qualitative analysis (e.g., thematic analysis): 2 • Content analysis: 2 • Analyses to examine impact of adaptations on various outcomes (e.g., regression analysis): 2 • Other: 1 research program 3 more methods: Causal loop modeling, Member checking with local advisory board, Configurational Comparative Methods (CCM) and Qualitative Comparative Analysis (QCA) • Not yet decided: 2 • Analyses to examine differences between adaptation(s) and outcome(s) (e.g., t-tests, ANOVA, MANOVA): 0 • • Rapid ethnographic analysis: 0

**Table 3B:** Operationalization of methods used to document adaptations in the 8 ACCSIS research programs.

Methods to document adaptation data	Operationalization of method	Who collects the data?	From whom data is being collected	Type of data collected	Frequency for data collection	Adaptation information collected
What methods are used to collect the adaptation data?	How do you operationalize this method?	Researcher(s)/Research Team Implementation partners Other	Researcher(s)/Research Team Implementation partners Community member/Patient Other	QUAL QUANT BOTH	Real time Weekly Bi-weekly Monthly Quarterly Variable (as needed or feasible/*ad hoc*) Other	ADAPT TYPE: PLAN: DESC: ELEMENT: SOURCE: CHANGE TYPE: WHEN: WHO: WHY: IMPACT: BASIS:
Periodic reflections for research team and/or implementation partners ACCSIS Programs: *n* = 5 (6 methods) Arizona, New Mexico, North Carolina (2), Oregon, San Diego	Combination of weekly and monthly in person and virtual meetings with navigators at clinical facilities, research team, and key personnel at all sites Research team reviewed research project and identified adaptations Periodic reflections are intended to provide an opportunity for reflecting on implementation and adaptations on a regular basis. At the beginning of standing monthly (previously bimonthly) meetings with partner clinic staff, the CA teams asks about 3–5 semi-structured reflection questions developed by the team. Two SCORE members then take notes on the clinic's response and, for the rest of the meeting, listen for language about adaptions and note down any topics appropriately. These notes are abstracted into a spreadsheet organized by reflection question and a subset of FRAME domains. Because our project is centralized, research team members are also implementers. Reflections with the research team focus on each intervention component (Registry, Mailed FIT, FIT+ to Colonoscopy) and allow the team to collectively reflect on and develop shared understanding of implementation activities and adaptations Qualitative analyst conducts monthly periodic reflection with practice facilitators and several supplemental periodic reflections with the whole research team at key points in the study. Qualitative analyst solicits topics from research team prior to session. Sessions are conducted via zoom, recorded, and transcribed During regular weekly team meetings, including the research team and research and implementation facilitation organization, discussions about challenges and potential solutions and related adaptations are conducted. Discussion is led by the implementation science specialist.	Researcher(s)/Research Team: 5 (6 NC) Implementation partners Other	Researcher(s)/Research Team: 5 Implementation partners: 2 Community member/Patient Other: 1 (Research and implementation facilitation partner)	QUAL: 5 QUANT: BOTH: 1	Real time: 1 Weekly: Bi-weekly: 1 Monthly: 2 Quarterly Variable (as needed or feasible/*ad hoc*): 3 Other: 1 (key time points in study)	ADAPT TYPE: 5 PLAN: 3 DESC: 6 ELEMENT: 5 SOURCE: 5 CHANGE TYPE: 4 WHEN: 5 WHO: 5 WHY: 6 IMPACT: 5 BASIS: 1 OTHER: 1
Review of meeting minutes and agendas ACCSIS Programs: *n* = 5 Appalachia, New Mexico, North Carolina, Oregon, San Diego	Project team members review minutes from monthly and quarterly clinic check-in calls to review any updates, including adaptations. Detailed notes for the meetings with the implementing partners (i.e., multisector action team) at the participating healthcare facilities are captured. All meeting notes are reviewed for strategies chosen and adaptation made. Using meeting notes from all project meetings, team will retrospectively code and analyze meeting notes, with specific focus on FRAME topics. Project manager and practice facilitators take notes to summarize meetings with health plan partners and clinic staff. Review of weekly meeting minutes, attended by the research team and the research and implementation facilitation organization, and meeting agendas to identify and capture adaptations.	Researcher(s)/Research Team: 5 Implementation partners Other	Researcher(s)/Research Team: 3 Implementation partners: 4 Community member/Patient Other: 1 (Research and implementation facilitation partner)	QUAL: 3 QUANT: BOTH: 2	Real time: Weekly: 1 Bi-weekly: 1 Monthly: 4 Quarterly: 2 Variable (as needed or feasible/*ad hoc*): 2 Other:	ADAPT TYPE: 3 PLAN: 2 DESC: 5 ELEMENT: 4 SOURCE: 4 CHANGE TYPE: 4 WHEN: 4 WHO: 4 WHY: 5 IMPACT: 4 BASIS: 4
Interviews with implementers ACCSIS Programs: *n* = 3 North Carolina, Oregon, San Diego	Interviews with implementers are intended to gather interviewee perspectives on implementation and adaptations. The ACCSIS-NC (SCORE) Capturing Adaptations (CA) team identifies key clinic partners and research staff who are directly involved with the intervention. Two CA team members facilitate the interview using a semi-structured interview guide based on the FRAME. Interviews are audio-recorded and transcribed. Semi-structured qualitative interviews with health plan and clinic partners. Research team prepares an interview guide and conducts the interviews in person or via zoom. Sessions are recorded and transcribed. A series of semi-structured interviews will be conducted mid-implementation and at the end of implementation with CHC stakeholders (e.g., Care Coordinators, CHC leaders, and clinicians). The interviews will include adaptation related questions.	Researcher(s)/Research Team: 3 Implementation partners: Other:	Researcher(s)/Research Team: 1 Implementation partners: 3 Community member/Patient: Other:	QUAL: 3 QUANT: BOTH:	Real time: Weekly: Bi-weekly: Monthly: Quarterly: Variable (as needed or feasible/*ad hoc*): 1 Other: 2 (Baseline/after year 1 implementation/post implementation Mid-implementation/end of implementation)	ADAPT TYPE: 2 PLAN: 2 DESC: 2 ELEMENT: 2 SOURCE: 3 CHANGE TYPE: 2 WHEN: 3 WHO: 2 WHY: 3 IMPACT: 3 BASIS: 2
Adaptations tracking at clinical site ACCSIS Programs: *n* = 3 Appalachia, Chicago, Oklahoma	Clinic champions describe any planned or unplanned adaptations during monthly and quarterly check-in calls. Track the intervention adaptation at each partner health system based on five domains: Service setting Target audience Mode of delivery Cultural Core component Navigator tracking log is used to record number of FIT kits mailed/returned at each clinic per month, how the FIT kits were distributed and returned (e.g., by mail, at the clinic, etc). This allows us to determine how these numbers vary when adaptations are made, including standing orders for FIT tests, distribution of FIT tests at COVID-19 vaccine and influenza vaccine events, mailed FIT kits with return envelopes, and a monthly lottery for a $20 gift card for returning FIT kits at one site.	Researcher(s)/Research Team: 3 Implementation partners: 1 Other:	Researcher(s)/Research Team: Implementation partners: 3 Community member/Patient: Other:	QUAL: QUANT: 1 BOTH: 2	Real time: Weekly: Bi-weekly: Monthly: 2 Quarterly: 1 Variable (as needed/*ad hoc*): Other:	ADAPT TYPE: 2 PLAN: 1 DESC: 2 ELEMENT: 2 SOURCE: 2 CHANGE TYPE: 2 WHEN: 2 WHO: 2 WHY: 2 IMPACT: 2 BASIS: 2
Review clinic contact logs and field notes ACCSIS Programs: *n* = 2 Chicago, Oregon	Feedback from implementer at partner site Facilitators complete field note forms in REDCap following clinic encounters. Forms include open and closed ended questions.	Researcher(s)/Research Team: 2 Implementation partners: Other:	Researcher(s)/Research Team: 1 Implementation partners: 2 Community member/Patient: Other:	QUAL: 1 QUANT: BOTH: 1	Real time: 1 Weekly: Bi-weekly: Monthly: Quarterly: Variable (as needed/*ad hoc*): 1 Other:	ADAPT TYPE: 2 PLAN: 1 DESC: 1 ELEMENT: 1 SOURCE: 1 CHANGE TYPE: 1 WHEN: 1 WHO: 1 WHY: 2 IMPACT: BASIS: 1
Informal check-in with clinical or community partners ACCSIS Programs: *n* = 2 Appalachia, San Diego	Clinic champions describe any planned or unplanned adaptations during progress check-ins with project manager. The research and implementation facilitation organization meets biweekly with each participating health center to discuss implementation and related challenges.	Researcher(s)/Research Team: 1 Implementation partners: Other: 1 (Research and implementation facilitation partner)	Researcher(s)/Research Team: Implementation partners: 2 Community member/Patient: Other:	QUAL: 1 QUANT: BOTH: 1	Real time: Weekly: Bi-weekly: 1 Monthly: Quarterly: Variable (as needed or feasible/*ad hoc*): 1 Other:	ADAPT TYPE: 1 PLAN: 1 DESC: 2 ELEMENT: 2 SOURCE: 1 CHANGE TYPE: 2 WHEN: 2 WHO: 2 WHY: 2 IMPACT: 2 BASIS: 2 OTHER: 1
Member-checking of data for missing adaptations and accuracy of data ACCSIS Programs: *n* = 2 Oregon, San Diego	Adaptations are logged into a tracking spreadsheet and reviewed for accuracy with project leadership team (project manager, PIs, lead analysts). All adaptations document in the real-time adaptation database are presented to the research team and research and implementation facilitation organization to review and confirm details of adaptation and identify missing adaptations.	Researcher(s)/Research Team: 2 Implementation partners: Other:	Researcher(s)/Research Team: 2 Implementation partners: 1 Community member/Patient: Other: 1 (Research and implementation facilitation partner)	QUAL: QUANT: BOTH: 2	Real time: Weekly: Bi-weekly: Monthly: Quarterly: Variable (as needed or feasible/*ad hoc*): 1 Other: 2 (key time points in the study, semi-annually)	ADAPT TYPE: 1 PLAN: 1 DESC: 2 ELEMENT: 2 SOURCE: 1 CHANGE TYPE: 2 WHEN: 2 WHO: 2 WHY: 2 IMPACT: 2 BASIS: 2 OTHER: 1
Real-time adaptation tracking database	Adaptation data is submitted by the team and manually recorded on a REDCap survey to enter into the adaptation-tracking database.	Researcher(s)/Research Team: 1 Implementation partners: Other: 1 (Research and implementation facilitation partner)	Researcher(s)/Research Team: 1 Implementation partners: 1 Community member/Patient: Other: 1 (Research and implementation facilitation partner)	QUAL: QUANT: BOTH: 1	Real time: 1 Weekly: Bi-weekly: Monthly: Quarterly: Variable (as needed or feasible/*ad hoc*): Other:	ADAPT TYPE: PLAN: DESC: 1 ELEMENT: 1 SOURCE: 1 CHANGE TYPE: 1 WHEN: 1 WHO: 1 WHY: 1 IMPACT: 1 BASIS: 1 OTHER: 1
Reminder email about adaptations to research team and partners	The Project Manager disseminates biweekly reminder email sent to the research team and the research and implementation facilitation organization.	Researcher(s)/Research Team: 1 Implementation partners: Other:	Researcher(s)/Research Team: 1 Implementation partners: Community member/Patient: Other: 1 (Research and implementation facilitation partner)	QUAL: 1 QUANT: BOTH:	Real time: Weekly: Bi-weekly: 1 Monthly: Quarterly: Variable (as needed or feasible/*ad hoc*): Other:	ADAPT TYPE: PLAN: DESC: 1 ELEMENT: SOURCE: CHANGE TYPE: WHEN: WHO: WHY: IMPACT: BASIS: OTHER: 1

QUAL, qualitative data; QUANT, quantitative data.

ADAPT TYPE—Type of adaptation (e.g., change in process, strategy, intervention, etc.).

PLAN—Adaptation was planned or unplanned.

DESC—Description of adaptation.

ELEMENT—Elements of the program were changed (e.g., setting, format, personnel, etc.).

SOURCE—Who provided information about the adaptation.

CHANGE TYPE—Type of change was made (e.g., tailoring, adding component, etc.)?.

WHEN—When adaptation was made.

WHO—Who initiated the adaptation.

WHY—Why the adaptation was made.

IMPACT—What the impact of the adaptation was.

BASIS—What the basis for the change (e.g., vision or values, framework or theory, staff or patient knowledge, etc.) was.

Nine unique approaches were used to *document* adaptations (Table 3A). RPs used an average of three methods to capture adaptations with a range of one to seven methods. The most used approaches to document adaptations were periodic reflections (22) for research team and implementation partners and review of meeting minutes and agendas (five RPs for each). Additional methods included interviews with implementers and adaptation tracking at clinical sites (three RPs for each), and review of clinical contact logs and field notes, informal check-ins with clinical or community partners, and member-checking of data for missing adaptations and accuracy (two for each). One RP also used a real-time adaptation tracking database, and one used reminder emails about adaptations to research team and partners.

Programs reported nine different analytic methods used to guide the analysis of adaptations (Table 3A). Individual RPs reported on average two analytic methods with a range of one to six. Most frequently reported methods included rapid qualitative analysis and descriptive statistics (three RPs for each) followed by mixed-methods analysis, standard (in-depth) qualitative analysis, content analysis, and analyses to examine the impact of adaptations on various outcomes (two for each). One RP also reported three additional innovative methods for analysis: causal-loop modeling (visual representation of interconnected relationships among key variables) (23), member-checking with a local advisory board (verifying findings with community members/research participants), and configurational comparative methods and qualitative comparative analysis (24). Two programs were undecided about methods for analysis.

All documentation methods used the research team to gather documentation information; two methods involved using the implementation partner or research and implementation facilitation partner as well (adaptation tracking at the clinical site, real-time adaptation tracking database). Roles within the research team included implementation specialists and program managers. Most methods allowed for the collection of adaptation information from the research team and implementation partners (i.e., clinical staff and providers at partner health centers). Only two methods were used to gather information from implementation partners exclusively (adaptation tracking at clinical site and informal check-in with clinical or community partners). Most methods involved collecting both qualitative and quantitative data; two involved the collection of only qualitative data (interviews with implementers, reminder email about adaptations to research teams and partners). Programs varied in how they operationalized each method in terms of frequency of data collection and adaptation information collected (Table 3B).

### 3.3 Characteristics of adaptations

Table 4 provides an overview of adaptations reported by the eight ACCSIS programs. A detailed description of each adaptation is provided in Additional File S3. A total of 96 adaptations were reported by the eight RPs as examples of the types of adaptations implemented during the initial study period. The number of adaptations reported across the RPs varied, with one program reporting as many as 22 adaptations and another reporting only 3.

**Table 4:** Summary characteristics of the 96 adaptations reported across the 8 ACCSIS research programs.

Adaptation characteristics	Pre-implementation	Early-implementation	Mid-implementation	Overall
Timing of adaptation				
*When was the adaptation first implemented?*	68% (65)	26% (25)	6% (6)	100% (96)
Adaptation elements^a^ Which elements were primarily changed?				
The setting	3% (2)	8% (2)	—	4% (4)
The format and/or how the intervention is presented	75% (49)	64% (16)	50% (3)	71% (68)
Personnel involved	14% (9)	16% (4)	50% (3)	17% (16)
The target population	3% (2)	4% (1)	—	3% (3)
Other	6% (4)	12% (3)	—	7% (7)
Adaptation type^a^ Which were the primary type of changes involved?				
Tailoring to individuals	17% (11)	—	—	11% (11)
Adding a component	18% (12)	56% (14)	67% (4)	31% (30)
Removing a component	8% (5)	—	—	5% (5)
Condensing a component	3% (2)	4% (1)	—	3% (3)
Extending a component	6% (4)	8% (2)	—	6% (6)
Substituting for a component	22% (14)	16% (4)	—	19% (18)
Changing the order of components	3% (2)	—	—	2% (2)
Integrating with other programs we are doing	3% (2)	8% (2)	—	4% (4)
Repeating a component	—	—	—	—
Loosening the structure or protocol	12% (8)	—	—	8% (8)
Otherwise changing the intervention	17% (11)	8% (2)	33% (2)	16% (15)
Components adapted^b^ Which components of your research program were affected by the adaptation?				
Mailed FIT process	52% (34)	24% (6)	17% (1)	43% (41)
Care coordination process/Navigation	34% (22)	40% (10)	33% (2)	35% (34)
Research logistics (IRB, language translation, incentives)	9% (6)	12% (3)	—	9% (9)
Study design and data collection and analysis process	8% (5)	16% (4)	—	9% (9)
Other	2% (1)	24% (6)	50% (3)	10% (10)
Adaptation reason^a^ Which were the primary reasons behind the adaptation?				
To increase the number or type of patients contacted (*reach*)	11% (7)	24% (6)	17% (1)	15% (14)
To enhance the impact or success of the intervention for all or important subgroups (*effectiveness*)	37% (24)	16% (4)	33% (2)	31% (30)
To make it possible to involve more teams, team members or staff (*adoption*)	3% (2)	12% (3)	17% (1)	6% (6)
To make the intervention delivered more consistently; to better fit the CHC/clinic, clinician needs, patient flow or EHR; for practical reasons, to enhance feasibility (*implementation*)	43% (28)	20% (5)	33% (2)	36% (35)
To save money or other resources (implementation)	8% (5)	—	—	5% (5)
To institutionalize or sustain the intervention (*maintenance*)	2% (1)	—	—	1% (1)
To respond to external pressures or policy	25% (16)	56% (14)	—	31% (30)
Other	3% (2)	—	—	2% (2)
COVID-19 pandemic relevance Was the adaptation in response to the COVID-19 pandemic?	26% (17)	60% (15)	50% (3)	36% (35)

^a^ Up to two options selected (% will not add up to 100).

^b^ Selected as many as relevant (% will not add up to 100).

CHC, community health center; EHR, electronic health record; IRB, institutional review board;.

Most of the adaptations occurred during the pre-implementation stage (68%), followed by early implementation (26%) and mid-implementation (6%). Most adaptations were made to the format or how the intervention is presented (71%), involved the addition of a component (31%), substitution for a component (19%), or otherwise changing the intervention (16%). Most also involved changes to the process for mailing stool-based CRC screening tests to patients (mailed Fecal Immunochemical Test) (43%) or to care coordination/navigation (35%). Adaptations were made to increase the consistency of delivery and fit of the program (implementation) (36%), to enhance the impact or success of the intervention for all or important subgroups (effectiveness) (31%), or to respond to external pressures (31%). Only 36% of the adaptations were attributed to the COVID-19 pandemic.

## 4 Discussion

To date, we have successfully established a shared vision of tracking adaptations across the ACCSIS initiative, guided by a series of workgroup meetings, review of the literature, and presentations by experts in the field. This vision included both agreement on the need for documenting and reporting adaptations across the consortium and defining adaptations for our purposes. RPs reported methods they used to track adaptations and documented and reported their programs' pre-, early-, and mid-implementation adaptations.

We synthesized adaptation definitions and documentation methods used across the eight RPs and described the significant variation in definitions, key personnel, and data collection and analytic methods used. We also tested the feasibility of collating information about adaptations across the RPs using a harmonized instrument identifying a small set of key characteristics of adaptations.

Adaptations were presented by RPs as complex adaptations that might have included more than one change. Similarly, we also noted variation in the number of reported adaptations across programs, possibly due to the diversity in definitions, that is, some programs documented more nuanced changes whereas others documented broader, bigger-picture changes. We kept reported adaptations as is (whether they encompassed one or a collection of adaptations), classifying them as one adaptation as defined by the RPs. However, future work could refine how adaptations are defined (for e.g., content vs. delivery adaptations) and apply that definition consistently across programs.

Throughout our process, we found it challenging to determine *what constitutes* an adaptation. Does scope matter? Does magnitude? For example, does tailoring patient-facing materials with a logo that includes a new community partner's name constitute an adaptation, or is it simply “standard practice” in study start-up? We wrestled with this example and others. In our discussions, we observed that often the perceived size or type of adaptation was not necessarily the best predictor of its importance (as perceived by the RPs) and that understanding the impact of the adaptation on implementation and effectiveness outcomes can be a better indicator of whether an adaptation (regardless of its magnitude) should be documented. In the context of the logo change example, a determination needs to be made if this modification was expected to lead to better implementation or effectiveness outcomes, such as the reach of the community. If yes, then it should be considered an adaptation and documented as such. If no meaningful change in outcomes is expected, then the change would not rise to the level of adaptation. As such, the same change could be seen as an adaptation or not depending on the study context. This determination took much discussion and time and underlies the need for greater guidance around adaptations documentation, such as criteria that can be applied each time the “is it an adaptation?” question arises. McCreight and colleagues describe a similar approach to determining whether a change should be documented as an adaptation (18). Although not within scope for this project, understanding the impact of adaptations on outcomes will be critical to advancing the study of adaptations within implementation science.

Despite agreement across the consortium to document and report adaptations, several RPs initially expressed some hesitancy to participate in the data collection process. This reluctance was largely related to time and effort involved in the process of tracking, documenting, and reporting the adaptations considering competing priorities. To a lesser extent, reluctance was due to the novelty of adaptations. Awareness that adaptations take place in planned interventions did not translate to deep understanding about or agreement with the importance of capturing adaptations. The limited time available for tracking and lack of ready tracking systems and tools served as barriers to embracing and executing adaptations tracking in practice, and thus are critical considerations when planning for documenting adaptations.

We offer additional considerations for documenting and sharing information about adaptations for groups such as consortia who are considering tracking adaptations across their respective partners. These suggestions highlight the importance of giving attention to adaptations, defining them *a priori*, and having simplified methods of data collection, tracking, analysis, and reporting.

### 4.1 Develop a shared approach to adaptations

It will be critical for research teams across sites and/or programs to have a shared vision of the role of adaptations and agree on the purpose of tracking adaptations for their specific project. At a minimum, this would include group discussions of the relevance of and rationale for adaptations to document, analyze, and report them, as well as consideration of how adaptations are defined and harmonized across programs. The vision could include agreement on a minimum number or type of adaptations to track or a specified period of tracking.

### 4.2 Clarify key intervention elements

We suggest that each entity define the intervention's key elements, both for the intervention and implementation strategies. When appropriate, we recommend harmonizing this across RPs. This will serve to develop a shared understanding of both components and strategies as a first step to identifying adaptations to each. Programs aiming to do this can consider applying a framework or process, such as the functions and forms approach (25, 26).

### 4.3 Utilize harmonized instruments

Likewise, agreement on the use of shared tools (e.g., an Excel matrix) will be critical for comparing adaptations across groups. Development of a harmonized instrument with agreed-upon constructs, sub-constructs, and their operational definitions will aid data collection from, and comparison across, multiple RPs.

### 4.4 Develop an analysis and dissemination plan

Finally, we recommend that groups give thought ahead of time about what to do with the adaptation information collected, including how to analyze it, how to share it, in what form, and with whom. Will the findings be used to inform scale-up? To promote sustainment? For shared learning, and if so with whom? How will the information get back to clinic and community partners, and how can they be helped to utilize the information? These questions and others are important to consider, plan for, and document, if the collection of adaptations is to serve a greater purpose beyond an academic exercise.

The work herein provides an understanding of how information about adaptations is documented across diverse settings, setting the stage for understanding how tracking and analyzing adaptations can inform implementation and interpretation of study results within and across research studies. Understanding adaptations across study sites can help trans-ACCSIS efforts to interpret findings across the consortium, maximizing learning. Variation in implementation is inevitable, and lack of understanding of the ways in which sites vary can interfere with interpreting results and limit shared learning. Likewise, this work underscores the importance of tracking adaptations within single sites as well—changes to interventions and implementation strategies are inevitable and ignoring them can interfere not only with analysis, but also with understanding impact, replication, or sustainment efforts.

One of the earliest efforts to document adaptations in a larger program was established in two consecutive iterations of the U.S. Department of Veterans Affairs-funded Quality Enhancement Research Initiatives Triple Aim and Quadruple Aim QUERI programs. In these programs, a similar system was set up to document adaptations across the research projects (17, 18, 27).

Parallel to our efforts to document adaptations systematically across RPs in the ACCSIS Program, other National Institutes of Health (NIH)-funded initiatives also engaged in such efforts, some of which are still in progress. Smith and colleagues (28) developed a novel systematic methodology (Longitudinal Implementation Strategies Tracking System; LISTS) to classify, document, and track the use of implementation strategies across three trials focused on improving symptom management as part of the NCI-funded Improving the Management of symptoms during and following Cancer Treatment (IMPACT) Consortium (28). We are also aware of ongoing efforts through the National Heart Lung and Blood Institute-funded Disparities Elimination through Coordinated Interventions to Prevent and Control Heart and Lung Disease (DECIPHeR) Alliance (29, 30) and the NCI-funded Exercise and Nutrition Interventions to Improve Cancer Treatment-Related Outcomes (ENICTO) Consortium (31–33) to establish cross-program adaptation documentation using a combination of the RE-AIM expanded FRAME (13) and the function and form matrices (25). Although beyond the current scope of our project, future endeavors should investigate which combination of methods for documenting adaptations provides the most meaningful information, as well as how to evaluate the impact of adaptations on a wide range of implementation outcomes.

Key strengths of our study included the use of multiple data sources, the development of a systematic, theory-driven approach applicable across multiple RPs, and engagement with diverse, partners in the context of the documentation process.

Our study has some important limitations. Practical considerations led us to select a subset of constructs from the FRAME framework (11). Applying the full framework, including conceptualizing and operationalizing the constructs and data collection and analysis, would not have been feasible given program staff time constraints. We selected what we believed to be the most pragmatic constructs but may have missed other constructs important to understanding adaptations across our programs. This decision proved to be a necessary balance between complete application and feasibility for collecting adaptation information which requires careful consideration in all research studies undertaking documentation of adaptations.

To further reduce burden, and in the spirit of piloting this work, we asked each RP to provide *examples* of adaptations made rather than a comprehensive list. The examples were intended as a first pass at understanding what adaptations were being made, how they were operationalized, and how they were being captured. As such, we cannot make any claims or assumptions about the entirety of adaptations made across programs. We cannot, for example, make inferences about the number of adaptations made by intervention stage (e.g., early, mid, late). While the decision to include sample adaptations was intentional (i.e., demonstrate feasibility and the methodology), this limits the generalizability of our findings and directs the primary focus on the methodology.

Although RPs had diverse definitions for adaptations, over time and through group discussions and multiple iterations of developing the tables, there was a natural process of harmonization. Thus, although the definitions provided in the tables reflect some diversity of thought, there is also potential “group think”; individual program variation specific to context may have been lost. However, programs shared their open and forthright thoughts about adaptations in their context, suggesting that program-specific ideas and practices remained evident throughout the adaptation data collection process. Paradoxically, the process of defining adaptations also helped programs reflect on the meaning and implication of adaptations and helped them develop a shared understanding of adaptations in their specific contexts, suggesting that settling on a definition, however tending toward the center, is also the result of careful consideration by individual programs.

Our analysis cannot address how adaptations to interventions and implementation strategies interact. This exploration is being specified in a parallel program and has not been used to inform the current analysis. Furthermore, the current paper is primarily focused on demonstrating the feasibility of documenting adaptations across multiple RPs and did not attempt to analyze the impact of adaptations on diverse implementation and effectiveness outcomes. While still relatively new to the field, a recent publication by Aschbrenner and colleagues discussed guidance on how to consider adaptation impact. Future work should incorporate the assessment of adaptation on key outcomes (34).

## 5 Conclusion

Our work provides an important contribution to understanding how adaptations are characterized and monitored in practical settings and illuminates the need for pragmatic approaches and multiple methods of capturing adaptations tailored to the priorities and resources of RPs and partners. This understanding can serve implementation scientists and community members alike in understanding the role and power of adaptations in their own work. Consideration should be given to the importance of monitoring adaptations, defining them *a priori*, and having simplified methods of data collection, tracking, analysis, and reporting. The approach presented in this paper can provide a template for future replication studies and adaptation tracking in other consortia.

## Data Availability

The original contributions presented in the study are included in the article/Supplementary Material, further inquiries can be directed to the corresponding author.
