# Supplementary material for: Documenting adaptations across the Accelerating Colorectal Cancer Screening and follow-up through Implementation Science research programs: methods and adaptation examples
Source: Front Health Serv. 2025 Sep 26;5:1613925. doi: 10.3389/frhs.2025.1613925 (PMC12511014; doi:10.3389/frhs.2025.1613925)
Supplement: Supplementary file 1 [file Datasheet1.pdf]

frontiers

Supplementary Material

1 Supplementary Data

1.1 Supplementary Tables

Additional file 1: Description of ACCSIS Research Programs

ACCSIS Research Program	Region	Target Population and Health System/Clinic Type
ACCSIS San Diego	San Diego County, California	Age 50 to 75 years, not up-to-date, served by 1 of 3 FQHC systems
ACCISIS Oregon	Rural and frontier communities of Oregon	Age 50-74 Medicaid and dual (Medicaid-Medicare) recipients
ACCSIS Chicago	Cook County, Illinois, with an emphasis on Chicago	Age 50-74 racial/ethnic minority and low-income populations.
ACCSIS Appalachia	12 Appalachian counties in Ohio and Kentucky	Age 50-74 rural and medically underserved
ACCSIS North Carolina	Regions in North Carolina with high CRC burden and low CRC screening	Age 50-74, not up-to-date, served by 1 of 2 FQHC systems
ACCSIS Arizona	Largely rural AI/AN communities in Arizona	AI/AN aged 50-75 years at average risk for CRC without exclusions
ACCSIS New Mexico	Largely rural AI communities in the Albuquerque Area Southwest Tribal Epidemiology Center (AASTEC) Service Area (NM and TX)	AI aged 45-75 years at average risk for CRC served by tribally-operated healthcare facilities
ACCSIS Oklahoma	Rural and urban AI communities in Oklahoma	AI aged 45-75 years at average risk for CRC, served by 1 Indian Health Service, 1 tribal, or 1 urban AI healthcare facility

AI/AN =American Indian/Alaska Native; CRC = colorectal cancer; FQHC = Federally Qualified Health Center.

Additional file 2: Data collection instrument for methodologies of adaptation documentation and analysis for the eight ACCSIS Research Programs

ACCSIS Adaptations Documentation Methods

Version 2.23.2022

The purpose of this survey is to collect information about methods that are being used by each ACCSIS Research project. The information from this survey will be included in the trans-ACCSIS adaptation manuscript to be submitted to the special issue of *Frontiers in Health Services* and *Frontiers in Public Health*. Please provide information as complete as you can. Please note that we are not intending to suggest that all these data need to be collected by all research projects. Instead, we are identifying all methods and data elements that are collected across the projects.

Please feel free to invite all relevant members of your team to inform the information you enter into this survey. Please complete one survey per ACCSIS research project.

If you have questions about any of the fields, please email Borsika Rabin (barabin@health.ucsd.edu).

Thank you!!

Tool Field Name	Notes
**1. ACCSIS site:**	ENTER NAME OF ACCSIS Research Project title
**2. Person(s) responding:**	Provide names of those involved with completing this survey
Adaptation documentation methodology	
**3. How did you define adaptations?**<br>Your definition edited by us:	Please confirm this is correct or provide an edited version of the adaptation here:
**4. Who decides about what is considered an adaptation in your ACCSIS program? (Select all that apply)**<br>___ Researcher(s)/Research Team<br>___ Implementation partners (clinic administrators, program managers, clinicians, healthcare staff, etc.)<br>___ Community members/Patients<br>___ Other:<br>Please write in: ______________________________	Please place an X in front of all that applies.
**5. Do you use any theoretical framework for guiding your adaptation documentation? (Select all that apply)**<br>___ FRAME<br>___ FRAME-IS<br>___ Other:<br>Please write in: ______________________________<br>___ We are not using a framework for documenting adaptations	
**6. What methods are used to collect the adaptation data? (Select all that apply)**<br>___ Interviews with implementers<br>___ Periodic reflections for research team and/or implementation partners<br>___ Adaptations tracking at clinical site<br>___ Real-time adaptation tracking database<br>___ Reminder email about adaptations to research team and partners<br>___ Review clinic contact logs and field notes<br>___ Review of meeting minutes and agendas<br>___ Review of calendar entries	Please indicate all the methods you use to collect information about adaptations. We will ask you to answer a set of questions about each method in the next section.

Tool Field Name	Notes
___ Informal check-in with clinical or community partners<br>___ Member-checking of data for missing adaptations and accuracy of data<br>___ Other: write in<br>Please write in: ______________________________	
**7. How will you analyze/are you analyzing the adaptation information? Please consider your analytic approaches across all adaptation documentation methods.**<br>**(Select all that apply)**<br>___ Standard/traditional (in-depth) qualitative analysis (e.g., thematic analysis)<br>___ Rapid qualitative analysis<br>___ Rapid ethnographic analysis<br>___ Descriptive statistics (e.g., mean, median, mode, frequency, range)<br>___ Content analysis<br>___ Mixed methods analysis. (any kind)<br>___ Analyses to examine differences between adaptation(s) and outcome(s) (e.g., t-tests, ANOVA, MANOVA)<br>___ Analyses to examine impact of adaptations on various outcomes (e.g., regression analysis)<br>___ Not yet decided<br>___ Other:<br>Please write in: ______________________________	
**8. Method 1: [first method selected in Question 6]**<br>**Name of method from Question 6:**<br>___ ____	For each method you selected in Question 6, please provide information about:<br>A. How do you operationalize this method?<br>B. Who collects the data?<br>C. From whom the data are collected?<br>D. What type of data are collected?<br>E. How frequently data are collected?<br>F. What information about adaptations is collected?
**A. How do you operationalize this method?**<br>Please describe this briefly.	Please provide a brief description what this method looks like in your research project.
**B. Who collects the data? (Select all that apply)**<br>• Researcher(s)/Research TeamWho (role)? ______________________________<br>• Implementation partners (clinic administrators, program managers, clinicians, health care staff, etc.)<br>Who (role)? ______________________________<br>• Other:<br>Who (role)? ______________________________	
**C. From whom are the adaptation data collected? (Select all that apply)**<br>___ Researcher(s)/Research Team<br>Who (role)? ______________________________<br>___ Implementation partners (e.g., clinic administrators, program managers, clinicians, healthcare staff, navigator, QI manager, etc.)<br>Who (role)? ______________________________<br>___ Community member/Patient<br>Who (role)? ______________________________<br>___ Other:<br>Who (role)? ______________________________	For this question, please specify <u>ALL types</u> of roles relevant as a source of adaptation information.
**D. What types of data are collected about the adaptation? (Select all that apply)**<br>___ Qualitative<br>___ Quantitative	

Tool Field Name	Notes
**E. How frequently are adaptation data collected?**<br>___ Real time<br>___ Weekly<br>___ Biweekly<br>___ Monthly<br>___ Quarterly<br>___ Variable (as needed/ad hoc)<br>___ Other (write in)<br>Please write in: ______________________________	
**F. What information is collected about the adaptations? (Select all that apply)**<br>___ Type of adaptation (e.g., change in process, strategy, intervention)<br>___ Adaptation was planned or unplanned<br>___ Description of adaptation<br>___ Who provided information about the adaptation<br>___ Elements of the program were changed (e.g., setting, format, personnel, etc.)<br>___ Type of change was made (e.g., tailoring, adding component)?<br>___ When adaptation was made<br>___ Who initiated the adaptation<br>___ Why the adaptation was made<br>___ What the impact of the adaptation was<br>___ What the basis for the change (e.g., vision or values, framework or theory, staff or patient knowledge) was<br>___ Other:<br>Please write in: ______________________________	Please select characteristics of adaptations that you collect using the method.
**9. Method 2: [second method selected in Question 6]**<br>**Name of method from Question 6:**<br>___ ____<br>**[If only used one method, you are done!!]**	For each method you selected in Question 6, please provide information about:<br>G. How do you operationalize this method?<br>H. Who collects the data?<br>I. From whom the data are collected?<br>J. What type of data are collected?<br>K. How frequently data are collected?<br>L. What information about adaptations is collected?
**A. How do you operationalize this method?**<br>Please describe this briefly.	Please provide a brief description what this method looks like in your research project.
**B. Who collects the data? (Select all that apply)**<br>• Researcher(s)/Research Team<br>Who (role)? ______________________________<br>• Implementation partners (clinic administrators, program managers, clinicians, healthcare staff, etc.)<br>Who (role)? ______________________________<br>• Other:<br>Who (role)? ______________________________	
**C. From whom are the adaptation data collected? (Select all that apply)**<br>___ Researcher(s)/Research Team<br>Who (role)? ______________________________<br>___ Implementation partners (e.g., clinic administrators, program managers, clinicians, healthcare staff, navigator, QI manager, etc.)<br>Who (role)? ______________________________<br>___ Community member/Patient<br>Who (role)? ______________________________<br>___ Other:<br>Who (role)? ______________________________	For this question, please specify <u>ALL types</u> of roles relevant as a source of adaptation information.

Tool Field Name	Notes
**D. What types of data are collected about the adaptation? (Select all that apply)**<br>___ Qualitative<br>___ Quantitative	
**E. How frequently are adaptation data collected?**<br>___ Real time<br>___ Weekly<br>___ Biweekly<br>___ Monthly<br>___ Quarterly<br>___ Variable (as needed/ad hoc)<br>___ Other (write in)<br>Please write in: ______________________________	
**F. What information is collected about the adaptations? (Select all that apply)**<br>___ Type of adaptation (e.g., change in process, strategy, intervention, etc.)<br>___ Adaptation was planned or unplanned<br>___ Description of adaptation<br>___ Who provided information about the adaptation<br>___ Elements of the program were changed (e.g., setting, format, personnel, etc.)<br>___ Type of change was made (e.g., tailoring, adding component, etc.)?<br>___ When adaptation was made<br>___ Who initiated the adaptation<br>___ Why the adaptation was made<br>___ What the impact of the adaptation was<br>___ What the basis for the change (e.g., vision or values, framework or theory, staff or patient knowledge, etc.) was<br>___ Other:<br>Please write in: ______________________________	Please select characteristics of adaptations that you collect using the method.
**10. Method 3: [third method selected in Question 6]**<br>**Name of method from Question 6:**<br>___ ____<br><br>**[If only used two methods, you are done!!]**	For each method you selected in Question 6, please provide information about:<br>M. How do you operationalize this method?<br>N. Who collects the data?<br>O. From whom the data are collected?<br>P. What type of data are collected?<br>Q. How frequently data are collected?<br>R. What information about adaptations is collected?
**A. How do you operationalize this method?**<br>Please describe this briefly.	Please provide a brief description what this method looks like in your research project.
**B. Who collects the data? (Select all that apply)**<br>• Researcher(s)/Research Team<br>Who (role)? ______________________________<br>• Implementation partners (clinic administrators, program managers, clinicians, healthcare staff, etc.)<br>Who (role)? ______________________________<br>• Other:<br>Who (role)? ______________________________	
**C. From whom are the adaptation data collected? (Select all that apply)**<br>___ Researcher(s)/Research Team<br>Who (role)? ______________________________<br>___ Implementation partners (e.g., clinic administrators, program managers, clinicians, healthcare staff, navigator, QI manager, etc.)<br>Who (role)? ______________________________	For this question, please specify <u>ALL types</u> of roles relevant as a source of adaptation information.

Tool Field Name	Notes
___ Community member/Patient<br>Who (role)? _______________________________<br>___ Other:<br>Who (role)? ________________________________	
**D. What types of data are collected about the adaptation? (Select all that apply)**<br>___ Qualitative<br>___ Quantitative	
**E. How frequently are adaptation data collected?**<br>___ Real time<br>___ Weekly<br>___ Biweekly<br>___ Monthly<br>___ Quarterly<br>___ Variable (as needed/ad hoc)<br>___ Other (write in)<br>Please write in: _______________________________	
**F. What information is collected about the adaptations? (Select all that apply)**<br>___ Type of adaptation (e.g., change in process, strategy, intervention, etc.)<br>___ Adaptation was planned or unplanned<br>___ Description of adaptation<br>___ Who provided information about the adaptation<br>___ Elements of the program were changed (e.g., setting, format, personnel, etc.)<br>___ Type of change was made (e.g., tailoring, adding component, etc.)?<br>___ When adaptation was made<br>___ Who initiated the adaptation<br>___ Why the adaptation was made<br>___ What the impact of the adaptation was<br>___ What the basis for the change (e.g., vision or values, framework or theory, staff or patient knowledge, etc.) was<br>___ Other:<br>Please write in: _______________________________	Please select characteristics of adaptations that you collect using the method.

[More than 3 Methods? Please copy question 10 with all subsection and complete them. Thank you!!]

Additional file 3: Sample adaptations and their characteristics as reported by the 8 ACCSIS Research Programs

The information in the following table was provided by ACCSIS programs and revised for clarity by study analysts. The final draft was validated by program staff. The prompts for data collection are listed below.

1. Which ACCSIS program reported the adaptation?

- Appalachia
- Arizona
- Chicago
- New Mexico
- North Carolina
- Oklahoma
- Oregon
- San Diego

2. Brief description of the adaptation that was made (open text)

3. Timing of adaptation: At which of the following points in the project was this change first made?

- Pre-implementation
- Early implementation
- Mid-implementation
- Late implementation
- Sustainment

4. Adaptation elements: Which of the following elements was primarily changed as part of the adaptation?

- The setting
- The format and/or how the intervention is presented
- Personnel involved
- The target population
- Other

5. Adaptation type: Which of the following was the primary type of change involved?

- Tailoring to individuals
- Adding a component
- Removing a component
- Condensing a component
- Extending a component
- Substituting for a component
- Changing the order of components
- Integrating with other programs we are doing
- Repeating a component
- Loosening the structure or protocol
- Otherwise changing the intervention

6. Adaptation reason: Which of the following was the primary reason behind this change?

- To increase the number or type of patients contacted (reach)
- To enhance the impact or success of the intervention for all or important subgroups (effectiveness)
- To make it possible to involve more teams, team members or staff (adoption)
- To make the intervention delivered more consistently; to better fit the CHC/clinic, clinician needs, patient flow or EHR; for practical reasons, to enhance feasibility (implementation)
- To institutionalize or sustain the intervention (maintenance)
- To respond to external pressures or policy
- To save money or other resources (implementation)
- Other

7. Was the adaptation in response to the COVID-19 pandemic?
Yes / No

8. Components: What are the elements of your intervention and/or implementation strategies that are key change agents in your program? (select all that apply)

- Mailed FIT process
- Care coordination or navigation
- Research logistics (IRB, language translation, incentives)
- Study design and data collection and analysis process

Other: describe

ACCSIS Program	Description	Timing	Elements	Type	Reason for adaptation	COVID-19 related?	Components
Appalachia	Mailed FIT discontinued & Cologuard referral implemented	Pre-implementation	The format and/or how the intervention is presented	Substituting for a component	To make the intervention delivered more consistently; to better fit the CHC/clinic, clinician needs, patient flow or EHR; for practical reasons (implementation)	No	Mailed FIT
Appalachia	Add mailed FIT, including customized primer letter and patient education materials	Pre-implementation	The format and/or how the intervention is presented	Adding a component	To increase the number or type of patients contacted (reach); to enhance the impact or success of the intervention for all or important subgroups (effectiveness)	No	Mailed FIT
Appalachia	Patient reminder letter for CRC screening in COVID adapted environment	Pre-implementation	The format and/or how the intervention is presented	Extending a component	To respond to external pressures or policy	Yes	Care coordination or navigation
Appalachia	Patient reminder letter for wellness visits, including CRC screening	Pre-implementation	The format and/or how the intervention is presented	Adding a component; integrating with other programs we are doing	To make the intervention delivered more consistently; to better fit the CHC/clinic, clinician needs, patient flow or EHR; for practical reasons, to enhance feasibility (implementation)	No	Care coordination or navigation
Appalachia	CRC risk assessment	Pre-implementation	The format and/or how the intervention is presented	Adding a component	To enhance the impact or success of the intervention for all or important subgroups (effectiveness)	No	Care coordination or navigation
Appalachia	Adapted CRC patient education materials and method of distribution	Pre-implementation	The format and/or how the intervention is presented	Tailoring to individuals	To make the intervention delivered more consistently; to better fit the CHC/clinic, clinician needs, patient flow or EHR; for practical reasons, to enhance feasibility (implementation)	No	Care coordination or navigation
Appalachia	Adopted social and digital media patient education and discontinued printed patient education materials	Pre-implementation	The format and/or how the intervention is presented	Substituting for a component	To respond to external pressures or policy	Yes	Care coordination or navigation
Appalachia	Provider education sessions adapted format and timing	Pre-implementation	The format and/or how the intervention is presented	Substituting for a component	To respond to external pressures or policy; to enhance the impact or success of the intervention for all or important subgroups (effectiveness)	Yes	Care coordination or navigation
Appalachia	Added and removed components to provider education plan	Pre-implementation	The format and/or how the intervention is presented	Adding a component; removing a component	To respond to external pressures or policy; to enhance the impact or success of the intervention for all or important subgroups (effectiveness)	No	Care coordination or navigation
Appalachia	Provider reminders printed instead of accessed in EHR	Pre-implementation	The format and/or how the intervention is presented	Substituting for a component	To make the intervention delivered more consistently; to better fit the CHC/clinic, clinician needs, patient flow or EHR; for practical reasons, to enhance feasibility (implementation)	No	Care coordination or navigation

ACCSIS Program	Description	Timing	Elements	Type	Reason for adaptation	COVID-19 related?	Components
Appalachia	Using population health nurses and case managers as patient navigators	Pre-implementation	Personnel involved	Substituting for a component.	To make the intervention delivered more consistently; to better fit the CHC/clinic, clinician needs, patient flow or EHR; for practical reasons, to enhance feasibility (implementation); to save money or other resources (implementation)	No	Care coordination or navigation
Appalachia	Discontinued patient navigation (for initial screening) via telephone	Pre-implementation	The format and/or how the intervention is presented	Removing a component	To make the intervention delivered more consistently; to better fit the CHC/clinic, clinician needs, patient flow or EHR; for practical reasons, to enhance feasibility (implementation)	No	Care coordination or navigation
Appalachia	Incentivized CRC screening	Pre-implementation	The format and/or how the intervention is presented	Adding a component	To enhance the impact or success of the intervention for all or important subgroups (effectiveness)	No	Care coordination or navigation; research logistics
Appalachia	Added low literacy FIT instructions to mailing	Pre-implementation	The format and/or how the intervention is presented	Adding a component	To enhance the impact or success of the intervention for all or important subgroups (effectiveness)	No	Mailed FIT
Appalachia	Shift to stool-based options during COVID	Pre-implementation	The format and/or how the intervention is presented	Adding a component	To respond to external pressures or policy	Yes	Care coordination or navigation
Appalachia	Patient reminder letter for CRC screening in COVID adapted environment	Pre-implementation	The format and/or how the intervention is presented	Substituting for a component	To respond to external pressures or policy; to increase the number or type of patients contacted (reach)	Yes	Care coordination or navigation
Arizona	Adapting an existing colorectal cancer screening and navigation protocol to American Indian communities.	Pre-implementation	The format and/or how the intervention is presented	Tailoring to individuals	To enhance the impact or success of the intervention for all or important subgroups (effectiveness)	No	Care coordination or navigation
Arizona	Prioritization of FIT as primary screening modality rather than primary screening colonoscopy	Early implementation	The format and/or how the intervention is presented	Adding a component	To enhance the impact or success of the intervention for all or important subgroups (effectiveness)	No	Care coordination or navigation
Arizona	Engagement of local community gastroenterologists for performing colonoscopies	Early implementation	Personnel involved	Adding a component	To enhance the impact or success of the intervention for all or important subgroups (effectiveness)	No	Care coordination or navigation
Arizona	Authorization of physician extenders, including project navigators, to order FITs	Early implementation	Personnel involved	Extending a component	To make it possible to involve more teams, team members or staff (adoption)	No	Care coordination or navigation
Arizona	Web-based rather than in-person navigator training and clinic staff in-services	Early implementation	The format and/or how the intervention is presented	Substituting for a component	To respond to external pressures or policy	Yes	Care coordination or navigation
Arizona	Expansion of the role of a project-specific REDCap database to log navigator interactions with patients and track colorectal cancer screening activities	Early implementation	The format and/or how the intervention is presented	Substituting for a component	To make the intervention delivered more consistently; to better fit the CHC/clinic, clinician needs, patient flow or EHR; for practical reasons, to enhance feasibility (implementation)	No	Study design, data collection, or analysis process

ACCSIS Program	Description	Timing	Elements	Type	Reason for adaptation	COVID-19 related?	Components
Arizona	EHR modifications made to improve EHR documentation of CRC screening at the patient and facility levels.	Early implementation	The format and/or how the intervention is presented	Otherwise changing the intervention	To make the intervention delivered more consistently; to better fit the CHC/clinic, clinician needs, patient flow or EHR; for practical reasons, to enhance feasibility (implementation)	No	Study design, data collection, or analysis process
Arizona	Hiring of a full-time data manager for the project	Early implementation	Personnel involved	Adding a component	To make the intervention delivered more consistently; to better fit the CHC/clinic, clinician needs, patient flow or EHR; for practical reasons, to enhance feasibility (implementation)	No	Study design, data collection, or analysis process
Arizona	Providing navigators with project-specific mobile phones and WiFi hotspot capability	Early implementation	Other	Adding a component	To respond to external pressures or policy; to make the intervention delivered more consistently; to better fit the CHC/clinic, clinician needs, patient flow or EHR; for practical reasons, to enhance feasibility (implementation)	Yes	Care coordination or navigation
Arizona	Deployment of project navigators to assist with COVID vaccination efforts	Early implementation	Personnel involved	Integrating with other programs we are doing	To respond to external pressures or policy	Yes	Other: Response to community needs and priorities
Arizona	Mailed FIT kits instead of distributing them during clinic visits	Early implementation	The format and/or how the intervention is presented	Adding a component	To respond to external pressures or policy; to increase the number or type of patients contacted (reach)	Yes	Care coordination or navigation
Arizona	Encouraging participating facilities to hire navigators devoted full time to the project rather than sharing project navigator responsibilities among multiple individuals	Mid-implementation	Personnel involved	Otherwise changing the intervention	To make the intervention delivered more consistently; to better fit the CHC/clinic, clinician needs, patient flow or EHR; for practical reasons, to enhance feasibility (implementation)	No	Other: Additional clinical sites. EHR modifications
Arizona	Document family history of colorectal cancer and access to US Postal Service using the REDCap database and EHR	Mid-implementation	The format and/or how the intervention is presented	Adding a component	To enhance the impact or success of the intervention for all or important subgroups (effectiveness)	No	Other: Additional clinical sites
Chicago	Training and support provided by existing program staff instead of hiring a patient navigator	Pre-implementation	Personnel involved	Substituting for a component	To institutionalize or sustain the intervention (maintenance); to make it possible to involve more teams, team members or staff (adoption)	Yes	Care coordination or navigation
Chicago	The introductory sessions were completed virtually via Zoom. HealthLinc introductory sessions for providers were facilitated on the clinic level, rather than in one general meeting for the health system.	Early implementation	The format and/or how the intervention is presented	Substituting for a component	To respond to external pressures or policy	Yes	Research logistics
Chicago	The staff education sessions were held virtually via Zoom	Early implementation	The format and/or how the intervention is presented	Substituting for a component	To respond to external pressures or policy	Yes	Research logistics

ACCSIS Program	Description	Timing	Elements	Type	Reason for adaptation	COVID-19 related?	Components
New Mexico	Added a instruction-sheet in addition to manufacturer's instructions for the mailed FIT	Pre-implementation	The format and/or how the intervention is presented	Adding a component	To enhance the impact or success of the intervention for all or important subgroups (effectiveness)	No	Mailed FIT
New Mexico	Virtual MAT meetings that included facilitation of intervention development and implementation	Early implementation	The format and/or how the intervention is presented	Adding a component	To respond to external pressures or policy	Yes	Mailed FIT; care coordination or navigation; research logistics; study design, data collection, or
New Mexico	Flyers about colorectal screening (small media) were distributed through community check-points by the guards to all cars going in and out of the communities	Early implementation	The format and/or how the intervention is presented	Adding a component	To respond to external pressures or policy; to increase the number or type of patients contacted (reach)	Yes	Analysis process, Care coordination or navigation; other: Education at the patient level
New Mexico	Drive through health fairs	Early implementation	The setting; the format and/or how the intervention is presented	Adding a component	To respond to external pressures or policy; to increase the number or type of patients contacted (reach)	Yes	Other: Education at the patient level
New Mexico	Virtual training for providers	Early implementation	The format and/or how the intervention is presented	Adding a component	To respond to external pressures or policy	Yes	Care coordination or navigation; other: Education at the provider level
New Mexico	Mailed FIT became necessary strategy during COVID as opposed to previously being an optional strategy	Early implementation	The format and/or how the intervention is presented	Adding a component	To respond to external pressures or policy	Yes	Mailed FIT process
New Mexico	Increase the frequency of trainings for providers	Early implementation	Other: increase frequency of provider training	Extending a component	To respond to external pressures or policy; to make it possible to involve more teams, team members or staff (adoption)	Yes	Care coordination or navigation; other: Education at the provider level
North Carolina	Scrubbing patient charts	Pre-implementation	The format and/or how the intervention is presented	Adding a component	To increase the number or type of patients contacted (reach)	No	Other: Query development to identify eligible patients; study design, data collection, or analysis process
North Carolina	Mailed FIT: Adapting mailed FIT materials in response to COVID-19	Pre-implementation	The format and/or how the intervention is presented	Tailoring to individuals; otherwise changing the intervention	To respond to external pressures or policy; to enhance the impact or success of the intervention for all or important subgroups (effectiveness)	Yes	Mailed FIT
North Carolina	Mailed FIT: Adapted our study design from the pilot to the trial in response to pilot findings.	Pre-implementation	The format and/or how the intervention is presented	Removing a component	To save money or other resources (implementation)	No	Mailed FIT

ACCSIS Program	Description	Timing	Elements	Type	Reason for adaptation	COVID-19 related?	Components
North Carolina	Mailed FIT: Adapting and improving envelope appearance to promote patients to open the FIT packet envelope	Pre-implementation	The format and/or how the intervention is presented	Otherwise changing the intervention	To enhance the impact or success of the intervention for all or important subgroups (effectiveness)	No	Mailed FIT
North Carolina	Mailed FIT: Changing contents and language of mailed FIT materials.	Pre-implementation	The format and/or how the intervention is presented	Adding a component; changing the order of components	To respond to external pressures or policy; to enhance the impact or success of the intervention for all or important subgroups (effectiveness)	Yes	Mailed FIT
North Carolina	Mailed FIT: Different workflows at each CHC required us to adapt the way FIT orders were processed.	Pre-implementation	The format and/or how the intervention is presented	Tailoring to individuals; integrating with other programs we are doing	To make the intervention delivered more consistently; to better fit the CHC/clinic, clinician needs, patient flow or EHR; for practical reasons, to enhance feasibility (implementation)	No	Mailed FIT
North Carolina	Navigation: Implementing motivational interviewing techniques to improve patient's experience with follow-up colonoscopy	Mid-implementation	The format and/or how the intervention is presented	Adding a component	To enhance the impact or success of the intervention for all or important subgroups (effectiveness)	No	Care coordination or navigation
North Carolina	Navigation: Adapting communication with patients who had FIT+ results to reflect changes to GI landscape surrounding COVID-19	Pre-implementation	The format and/or how the intervention is presented	Tailoring to individuals; otherwise changing the intervention	To respond to external pressures or policy; to enhance the impact or success of the intervention for all or important subgroups (effectiveness)	Yes	Care coordination or navigation
North Carolina	Navigation: Reducing call time between navigation calls to ensure patients received navigation support in between their follow-up colonoscopy consultation and procedure	Pre-implementation	The format and/or how the intervention is presented	Condensing a component	To enhance the impact or success of the intervention for all or important subgroups (effectiveness)	No	Care coordination or navigation
North Carolina	Mailed FIT: Changing contents and language of mailed FIT materials	Pre-implementation	The format and/or how the intervention is presented	Substituting for a component	To enhance the impact or success of the intervention for all or important subgroups (effectiveness)	No	Mailed FIT
North Carolina	Adapting to different levels of EHR access across CHCs, i.e., a CHC first requiring a SCORE staff member to be on site at the CHC in order to access patient records, to later allowing the SCORE staff to have remote EHR access	Pre-implementation	Personnel involved	Loosening the structure or protocol	To make the intervention delivered more consistently; to better fit the CHC/clinic, clinician needs, patient flow or EHR; for practical reasons, to enhance feasibility (implementation); to save money or other resources (implementation)	No	Care coordination or navigation; study design, data collection, or analysis process
Oklahoma	Added standing orders for FIT Kit distribution at one set of clinics	Pre-implementation	The format and/or how the intervention is presented	Adding a component	To increase the number or type of patients contacted (reach)	No	Mailed FIT

ACCSIS Program	Description	Timing	Elements	Type	Reason for adaptation	COVID-19 related?	Components
Oklahoma	One clinic added FIT kit distribution and return boxes in multiple location, e.g, parking lot drop box, pharmacy distribution and drop box, etc.	Pre-implementation	The setting; the format and/or how the intervention is presented	Extending a component	To increase the number or type of patients contacted (reach)	No	Mailed FIT
Oklahoma	Distribution of FIT kits at drive through COVID-19 and flu vaccine events	Early implementation	The setting	Adding a component	To respond to external pressures or policy; to increase the number or type of patients contacted (reach)	Yes	Mailed FIT
Oklahoma	Monthly gift card drawing for those who return FIT kit	Early implementation	The format and/or how the intervention is presented	Adding a component	To respond to external pressures or policy; to increase the number or type of patients contacted (reach)	Yes	Other: increase patient demand for screening
Oklahoma	Mailed FIT kits with postage paid return envelopes	Early implementation	The format and/or how the intervention is presented	Adding a component	To increase the number or type of patients contacted (reach)	Yes	Mailed FIT
Oklahoma	Public service announcements (social media, print media, etc.) to resume CRC screening and to get screened if aged 45 to 75	Mid-implementation	The format and/or how the intervention is presented	Adding a component	To respond to external pressures or policy; to increase the number or type of patients contacted (reach)	Yes, partially	Other: increase patient demand for screening
Oklahoma	Training a new cohort of navigators	Mid-implementation	Personnel involved	Otherwise changing the intervention	To make it possible to involve more teams, team members or staff (adoption)	Yes, partially	Care coordination or navigation
Oregon	Used REDCap for scrubbing patient lists, rather than an Excel tracking file.	Pre-implementation	The format and/or how the intervention is presented	Substituting for a component	To make the intervention delivered more consistently; to better fit the CHC/clinic, clinician needs, patient flow or EHR; for practical reasons, to enhance feasibility (implementation)	No	Mailed FIT
Oregon	Broadened identification and training of the navigators to include/support medical assistants, community health workers, referral coordinators, and front desk staff	Pre-implementation	Personnel involved	Extending a component	To make it possible to involve more teams, team members or staff (adoption); to make the intervention delivered more consistently; to better fit the CHC/clinic, clinician needs, patient flow or EHR; for practical reasons, to enhance feasibility (implementation)	No	Care coordination or navigation
Oregon	Clinic recruitment was done virtually via phone and web meetings, rather than in person.	Pre-implementation	The format and/or how the intervention is presented	Substituting for a component	To respond to external pressures or policy	Yes	Research logistics
Oregon	Clinic onboarding process simplified to accommodate clinic realities and competing priorities. This included collecting qualitative data related to clinic's ability to pull colorectal cancer data queries and shortening meetings from 1.5 hours to 30 minutes.	Pre-implementation	The format and/or how the intervention is presented	Loosening the structure or protocol	To respond to external pressures or policy; to make the intervention delivered more consistently; to better fit the CHC/clinic, clinician needs, patient flow or EHR; for practical reasons, to enhance feasibility (implementation)	Yes	Study design, data collection, or analysis process

ACCSIS Program	Description	Timing	Elements	Type	Reason for adaptation	COVID-19 related?	Components
Oregon	Allowed clinics to expand mailed FIT program beyond Medicaid/Medicare health plan targets. In this process clinic could contract directly with a mailed FIT vendor.	Pre-implementation	The target population	Extending a component	To increase the number or type of patients contacted (reach); to make the intervention delivered more consistently; to better fit the CHC/clinic, clinician needs, patient flow or EHR; for practical reasons, to enhance feasibility (implementation)	No	Mailed FIT
Oregon	Added a monthly meeting between health plans and clinics to facilitate program implementation and cross-site sharing rather than having all meetings occur 1:1 with clinics or with the health plans	Pre-implementation	The format and/or how the intervention is presented	Substituting for a component	To make the intervention delivered more consistently; to better fit the CHC/clinic, clinician needs, patient flow or EHR; for practical reasons, to enhance feasibility (implementation)	No	Mailed FIT; care coordination or navigation
Oregon	Broadened clinic data requirements to determine inclusion (allowed 2021 rather than 2019)	Pre-implementation	Other	Loosening the structure or protocol	To increase the number or type of patients contacted (reach)	Yes	Research logistics
Oregon	For one health plan, randomized health systems with multiple clinics as a single unit	Pre-implementation	Other	Loosening the structure or protocol	Other	No	Research logistics
Oregon	FIT tests go back to a central lab for processing with one health plan instead of labs affiliated with or in a clinic	Pre-implementation	Personnel involved	Substituting for a component	To respond to external pressures or policy; to make the intervention delivered more consistently; to better fit the CHC/clinic, clinician needs, patient flow or EHR; for practical reasons, to enhance feasibility (implementation)	No	Mailed FIT
Oregon	For one health plan, central lab notifies the clinics of FIT results via e-mail or fax (and also notifies their own care management team), rather than FITs being sent directly to clinics	Pre-implementation	Personnel involved	Substituting for a component	To make the intervention delivered more consistently; to better fit the CHC/clinic, clinician needs, patient flow or EHR; for practical reasons, to enhance feasibility (implementation)	No	Mailed FIT
Oregon	For one health plan partner, a centralized care management team reaches out by phone with results for positive FIT tests and tells patients to contact their provider	Pre-implementation	Personnel involved	Loosening the structure or protocol	To make the intervention delivered more consistently; to better fit the CHC/clinic, clinician needs, patient flow or EHR; for practical reasons, to enhance feasibility (implementation)	No	Mailed FIT
Oregon	One health plan decided to not mail an introduction letter centrally, and let clinics opt to mail one themselves	Pre-implementation	The format and/or how the intervention is presented	Removing a component	To make the intervention delivered more consistently; to better fit the CHC/clinic, clinician needs, patient flow or EHR; for practical reasons, to enhance feasibility (implementation)	No	Mailed FIT

ACCSIS Program	Description	Timing	Elements	Type	Reason for adaptation	COVID-19 related?	Components
Oregon	One health plan is not using the FIT kit insert created by Boot Camp Translation in our pilot (participatory stakeholder process)	Pre-implementation	The format and/or how the intervention is presented	Removing a component	To make the intervention delivered more consistently; to better fit the CHC/clinic, clinician needs, patient flow or EHR; for practical reasons, to enhance feasibility (implementation)	No	Mailed FIT
Oregon	Discussed changing one FIT mailing into two due to clinic staffing constraints, but kept one mailing due to cost concerns.	Early implementation	Other	Condensing a component	To save money or other resources (implementation); to make the intervention delivered more consistently; to better fit the CHC/clinic, clinician needs, patient flow or EHR; for practical reasons, to enhance feasibility (implementation)	No	Mailed FIT
Oregon	Clarified exclusion criteria to specify that health plans with other active mailed FIT programs can be included as long as populations are separate and no overlap mailing occurs.	Early implementation	The target population	Otherwise changing the intervention	To make it possible to involve more teams, team members or staff (adoption)	No	Research logistics
Oregon	One clinic decided to use a patient incentive for returned FITs	Early implementation	The format and/or how the intervention is presented	Adding a component	To enhance the impact or success of the intervention for all or important subgroups (effectiveness)	No	Mailed FIT
Oregon	One practice facilitator is assisting clinics with REDCap tracking of patients following FIT return	Mid-implementation	Personnel involved	Adding a component	To make the intervention delivered more consistently; to better fit the CHC/clinic, clinician needs, patient flow or EHR; for practical reasons, to enhance feasibility (implementation)	Yes	Mailed FIT
San Diego	Patients will only receive a FIT from the study.	Pre-implementation	The format and/or how the intervention is presented	Condensing a component	To enhance the impact or success of the intervention for all or important subgroups (effectiveness); to make the intervention delivered more consistently; to better fit the CHC/clinic, clinician needs, patient flow or EHR; for practical reasons, to enhance feasibility (implementation)	No	Mailed FIT
San Diego	Reminder call schedule added an additional text message, for a total of 2	Pre-implementation	The format and/or how the intervention is presented	Loosening the structure or protocol	To enhance the impact or success of the intervention for all or important subgroups (effectiveness); to save money or other resources (implementation)	No	Mailed FIT
San Diego	All participating Community Health Centers agreed to collaborate with a third party vendor.	Pre-implementation	Personnel involved	Otherwise changing the intervention	To make the intervention delivered more consistently; to better fit the CHC/clinic, clinician needs, patient flow or EHR; for practical reasons (implementation)	No	Mailed FIT
San Diego	Third party vendor will be responsible for the delivery of 5,000 mailed FIT kits and reminder calls.	Pre-implementation	The format and/or how the intervention is presented	Otherwise changing the intervention	To make the intervention delivered more consistently; to better fit the CHC/clinic, clinician needs, patient flow or EHR; for practical reasons (implementation)	No	Mailed FIT
San Diego	Possible lessening of data wave requests	Pre-implementation	The format and/or how the intervention is presented	Loosening the structure or protocol	To make the intervention delivered more consistently; to better fit the CHC/clinic, clinician needs, patient flow or EHR; for practical reasons (implementation)	No	Mailed FIT

ACCSIS Program	Description	Timing	Elements	Type	Reason for adaptation	COVID-19 related?	Components
San Diego	Corrected colorectal cancer Spanish primer for grammar error	Pre-implementation	The format and/or how the intervention is presented	Tailoring to individuals	To enhance the impact or success of the intervention for all or important subgroups (effectiveness)	No	Mailed FIT
San Diego	Updated logo to reflect project partners	Pre-implementation	The format and/or how the intervention is presented	Tailoring to individuals	To enhance the impact or success of the intervention for all or important subgroups (effectiveness); to make the intervention delivered more consistently; to better fit the CHC/clinic, clinician needs, patient flow or EHR; for practical reasons (implementation)	No	Mailed FIT
San Diego	Revised colorectal cancer Spanish primer for readability	Pre-implementation	The format and/or how the intervention is presented	Tailoring to individuals	To enhance the impact or success of the intervention for all or important subgroups (effectiveness)	No	Mailed FIT
San Diego	Development of COVID-19 messaging (postcard) to include in the FIT kit package	Pre-implementation	The format and/or how the intervention is presented	Adding a component	To enhance the impact or success of the intervention for all or important subgroups (effectiveness); to respond to external pressures or policy	Yes	Mailed FIT
San Diego	Revised primer for layout and word choice	Pre-implementation	The format and/or how the intervention is presented	Tailoring to individuals	To enhance the impact or success of the intervention for all or important subgroups (effectiveness)	No	Mailed FIT
San Diego	Revised results letter for word choice	Pre-implementation	The format and/or how the intervention is presented	Tailoring to individuals	To enhance the impact or success of the intervention for all or important subgroups (effectiveness)	No	Mailed FIT
San Diego	Eligibility criteria minor changes	Pre-implementation	The format and/or how the intervention is presented	Otherwise changing the intervention	To make the intervention delivered more consistently; to better fit the CHC/clinic, clinician needs, patient flow or EHR; for practical reasons (implementation)	No	Mailed FIT
San Diego	Usual Care Assessment modifications including adding timeframe, items to capture clinic level usual care, and rearranged COVID-19-relevant section	Pre-implementation	The format and/or how the intervention is presented	Otherwise changing the intervention	To enhance the impact or success of the intervention for all or important subgroups (effectiveness)	Yes	Research logistics
San Diego	Changed format of care coordination trainings from in-person to virtual	Pre-implementation	The format and/or how the intervention is presented	Substituting for a component	To enhance the impact or success of the intervention for all or important subgroups (effectiveness)	Yes	Care coordination or navigation
San Diego	Train-the-trainer model, instead of training by research staff, was adopted to accommodate staff turnover and facilitate onboarding	Pre-implementation	The format and/or how the intervention is presented	Loosening the structure or protocol	To make the intervention delivered more consistently; to better fit the CHC/clinic, clinician needs, patient flow or EHR; for practical reasons (implementation); to respond to external pressures or policy	Yes	Care coordination or navigation

ACCSIS Program	Description	Timing	Elements	Type	Reason for adaptation	COVID-19 related?	Components
San Diego	Due to COVID-19 related supply issues, FIT kits were directly secured from a group purchasing organization instead of the Community Health Centers.	Pre-implementation	Other --> how resources needed for the implementation are secured	Otherwise changing the intervention: A change in the way the FIT resource was obtained by the clinic. Bulk/group order instead of individual orders by sites are being explored.	To respond to external pressures or policy	Yes	Mailed FIT
San Diego	Change in staff at Community Health Center	Pre-implementation	Personnel involved	Otherwise changing the intervention	To make the intervention delivered more consistently; to better fit the CHC/clinic, clinician needs, patient flow or EHR; for practical reasons, to enhance feasibility (implementation); to respond to external pressures or policy	No	Staffing/support/ resources
San Diego	FIT orders for two of the three Community Health Centers are placed after the FIT sample is received instead of at the time of the mailings.	Pre-implementation	Other --> Timing of FIT order.	Changing the order of components	To save money or other resources (implementation)	No	Research logistics
San Diego	A participating Community Health Center had a usual care site close.	Pre-implementation	The setting	Otherwise changing the intervention: Change in participating usual care site	Other	No	Study design, data collection, or analysis process
San Diego	One of the participating Community Health Centers requested to review and approve the final patient sample.	Pre-implementation	The target population	Otherwise changing the intervention: Changing the process of identifying the patient sample	To make the intervention delivered more consistently; to better fit the CHC/clinic, clinician needs, patient flow or EHR; for practical reasons, to enhance feasibility (implementation)	No	Study design, data collection, or analysis process
San Diego	English and Spanish instructions for both types of FIT kits used in the study were modified to better fit our Community Health Centers and the population they serve.	Pre-implementation	The format and/or how the intervention is presented	Tailoring to individuals	To make the intervention delivered more consistently; to better fit the CHC/clinic, clinician needs, patient flow or EHR; for practical reasons, to enhance feasibility (implementation)	No	Mailed FIT
San Diego	Usual care at sites was changed to include additional outreach via text message campaign (not hosted by research project).	Early implementation	The format and/or how the intervention is presented	Integrating with other programs we are doing	To enhance the impact or success of the intervention for all or important subgroups (effectiveness)	No	Study design, data collection, or analysis process
